# Evaluating renal injury characteristics in different rat models of hyperuricemia and elucidating pathological molecular mechanisms via serum metabolomics

**DOI:** 10.3389/fphar.2024.1433991

**Published:** 2024-09-02

**Authors:** Mengwen Liu, Jing Shen, Xuanshi Chen, Tuerxunayi Dawuti, Hui Xiao

**Affiliations:** ^1^ School of Public Health, Xinjiang Medical University, Urumqi, China; ^2^ Key Laboratory of Environmental Exposome, Xinjiang Medical University, Urumqi, China

**Keywords:** hyperuricemia, renal function, metabolomics, oxidative stress, MetOrigin

## Abstract

Hyperuricemia has emerged as a significant global health concern, closely associated with various metabolic disorders. The adverse effects frequently observed with current pharmacological treatments for hyperuricemia highlight the urgent need for reliable animal models to elucidate the disease’s pathophysiological mechanisms, thereby facilitating the development of safer and more effective therapies. In this study, we established three rat models of hyperuricemia using potassium oxonate, either alone or in combination with fructose and adenine. Each model exhibited distinct pathological changes, with the combination of potassium oxonate, fructose, and adenine causing significantly more severe damage to liver and kidney functions than potassium oxonate alone. Serum metabolomics analyses revealed profound dysregulation in the metabolic pathways of purine, pyrimidines, and glutathione, underscoring the pivotal role of oxidative stress in the progression of hyperuricemia. We identified key biomarkers such as orotidine, ureidosuccinic acid, uracil, and pseudouridine, which are associated with uric acid-induced damage to hepatic and renal systems. MetOrigin tracing analysis further revealed that differential metabolites related to hyperuricemia are primarily involved in host-microbiome co-metabolic pathways, particularly in purine metabolism, with bacterial phyla such as *Pseudomonadota*, *Actinomycetota*, and *Ascomycota* being closely linked to the critical metabolic processes of uric acid production. These findings not only enhance our understanding of the pathogenic mechanisms underlying hyperuricemia but also provide a robust experimental model foundation for the development of innovative treatment strategies.

## 1 Introduction

Hyperuricemia is a globally recognized health concern (https://www.medscape.com), characterized by elevated serum uric acid levels. Epidemiological studies have documented a rising prevalence and an earlier onset of hyperuricemia (Li et al., 2017; Li et al., 2021; Ito et al., 2020), largely attributable to lifestyle changes (Zhang et al., 2022a). In China, the prevalence increased from 11.1% in 2015–2016 to 14.0% in 2018–2019, with notable gender disparities (Zhang et al., 2022a). Similarly, data from the National Health and Nutrition Examination Survey (NHANES) in the United States indicated an increase from 19.1% in 1988–1994 to 21.5% in 2007–2008 (Zhu et al., 2011). This upward trend is evident in both developed and developing countries, reflecting broader societal changes such as urbanization, increased sedentary behavior (Hou et al., 2021), and the consumption of high-purine diets (Zhou et al., 2022).

The etiology of hyperuricemia is complex, influenced by multiple factors, particularly dietary patterns (Zhang et al., 2022a). High purine intake from foods such as seafood, meat, animal offal, and alcoholic beverages leads to increased urate production (Zhang et al., 2022a; Zhou et al., 2022). The metabolic conversion of fructose to fructose-1-phosphate reduces adenosine triphosphate (ATP) levels and raises adenosine monophosphate (AMP) levels, thereby promoting uric acid production (Zhang et al., 2022b). Additionally, the evolutionary loss of uricase (urate oxidase) in humans leads to the accumulation of monosodium urate crystals, predisposing individuals to hyperuricemia, gout, and kidney stones (Liu et al., 2023). Beyond these conditions (Dalbeth et al., 2021), hyperuricemia triggers systemic effects, including uric acid deposition in renal tissues, which activates macrophages and inflammatory responses, contributing to nephropathy and its strong association with chronic kidney disease (CKD) (Balakumar et al., 2020; Sharaf El Din et al., 2017). Moreover, hyperuricemia is closely linked with various metabolic disorders (Zheng et al., 2024; Yanai et al., 2021), including diabetes mellitus (Liu et al., 2018), cardiovascular diseases (Borghi et al., 2020), hypertension (Johnson et al., 2018), and metabolic syndrome (Niu et al., 2022), through mechanisms involving oxidative stress, endothelial dysfunction, and systemic inflammation (Gherghina et al., 2022).

Current pharmacological treatments for hyperuricemia, such as allopurinol and febuxostat, primarily aim to modulate uric acid synthesis, reabsorption, and excretion (Shi et al., 2024). However, these medications often result in adverse effects, including hepatotoxicity (Fontana et al., 2021), nephrotoxicity (Esposito et al., 2017), and hypersensitivity reactions (Yang et al., 2015), highlighting the need for safer and more effective therapies. Reliable and stable animal models are crucial for exploring the pathophysiological aspects of hyperuricemia and facilitating the development of preventative and therapeutic agents (Lu et al., 2019). Currently, hyperuricemia models primarily involve mice and rats, using techniques that either increase endogenous uric acid sources (e.g., adenine, fructose), decrease renal uric acid clearance (e.g., ethambutol), inhibit uricase activity (e.g., potassium oxonate), or employ genetic modifications (e.g., ABCG2 knockout) (Zhou et al., 2024). These methods, applied individually or in combination, offer substantial insights into urate metabolism (Liu et al., 2024; Pan et al., 2020).

The variability in the selection of chemicals, dosages, and administration routes for creating hyperuricemia models has been noted in the literature (Liu et al., 2024; Dhouibi et al., 2021). Evaluating these models based on single indices and time points poses challenges in comprehensively understanding the disease’s onset and progression, thereby complicating the assessment of the stability and effectiveness of different modeling techniques.

In this study, we employed potassium oxonate, alone or in combination with fructose and adenine, to induce hyperuricemia in rat models and assess its reversibility. Systematic monitoring at various time points allowed for the examination of the characteristics of different modeling methods in inducing hyperuricemia and their impacts on hepatic and renal functions. This approach establishes a solid foundation for animal models in various drug development contexts. Through serum metabolomics, we aim to identify the key metabolic pathways and biomarkers altered in hyperuricemia, thereby enhancing our understanding of its molecular pathophysiology and informing the development of new therapeutic targets.

## 2 Materials and methods

### 2.1 Chemical reagents

Fructose, potassium oxonate, and sodium carboxymethyl cellulose were procured from Shanghai Yuanye Biotechnology Co., Ltd. (Shanghai, China). Allopurinol and adenine were sourced from Shanghai Aladdin Biochemical Technology Co., Ltd. (Shanghai, China). High-purity LC-MS grade methanol and acetic acid were purchased from Thermo Fisher Scientific (Waltham, Massachusetts, United States), and LC-MS grade water was supplied by Merck (Darmstadt, Germany). Diagnostic kits for measuring uric acid, creatinine, urea, aspartate aminotransferase, and alanine aminotransferase levels were acquired from Shenzhen Myriad Biomedical Electronics Co., Ltd. (Shenzhen, China). The hematoxylin and eosin (H&E) staining kit for histological analysis was obtained from Beijing Solarbio Science and Technology Co., Ltd. (Beijing, China).

### 2.2 Animals

Specific pathogen-free (SPF) male Sprague-Dawley (SD) rats, weighing between 200 and 240 g, were obtained from the Laboratory Animal Center of Xinjiang Medical University. The rats were housed in a controlled environment with a 12-h light-dark cycle, at an ambient temperature of 22°C ± 2°C and relative humidity of 45% ± 5%. They were provided *ad libitum* access to water and standard rodent chow. All experimental protocols involving animals were conducted in accordance with ethical standards and guidelines for animal welfare. The study’s design, care, and use of the animals were reviewed and approved by the Animal Ethics Committee of Xinjiang Medical University (Approval No. IACUC-JT-20230110-9), ensuring adherence to the principles of ethical conduct in animal research.

### 2.3 Group information

To ensure consistency in experimental outcomes, tail-vein blood samples were collected for fasting serum uric acid (SUA) analysis on the 7th and 14th days of acclimation. Rats with abnormal SUA levels—too high, too low, or exhibiting significant fluctuations—were excluded to ensure the reliability of the experimental data. The remaining 42 SD rats, stratified based on body weight and fasting SUA levels, were then randomly assigned into seven groups (n = 6 per group) to ensure balanced representation.

**FIGURE 1:**
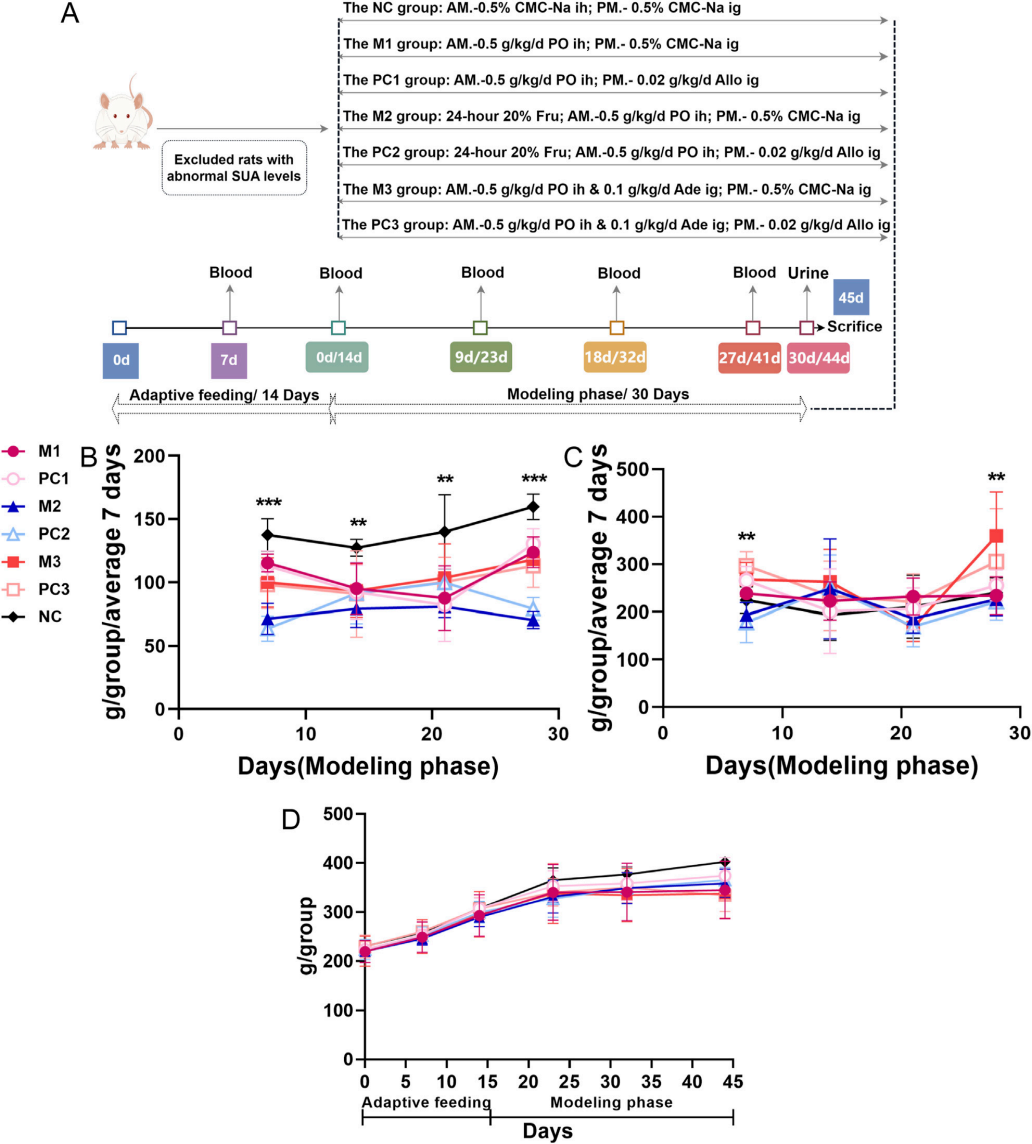
Temporal dynamics in food and water intake, and body weight in hyperuricemic rats. **(A)** the study protocol for assessing different hyperuricemia rat models; **(B)** food intake; **(C)** water intake; and **(D)** body weight. Data are depicted as mean ± SD for six rats per group (n = 6). Statistical differences between model and control groups were assessed using one-way ANOVA, with significance denoted by ^*^ *P* < 0.05, ^**^ *P* < 0.01, and ^***^ *P* < 0.001.

The group assignments and treatments were as follows (Figure 1A): the normal control group (NC), serving as the baseline for the study, received subcutaneous injections and oral doses of 0.5% sodium carboxymethyl cellulose (CMC-Na) solution. The first model group, the potassium oxonate (PO) group (M1), received 0.5 g·kg^−1^·d^−1^ of the PO solution via subcutaneous injections. In the second model, the PO + 20% fructose group (M2), rats received the same PO dosage and had *ad libitum* access to 20% fructose (Fru) water. The third model group, PO + adenine (M3), involved administering PO injections alongside oral doses of 0.1 g·kg^−1^·d^−1^ adenine (Ade). Additionally, three positive control groups (PC1, PC2, and PC3), each corresponding to one of the model groups, were treated with a daily oral dose of 0.02 g·kg^−1^·d^−1^ allopurinol (Allo) in the afternoon to assess the reversibility and therapeutic potential of the treatments against the induced hyperuricemia. The molding period was set at 30 days, allowing for the observation of both the acute and chronic effects of the hyperuricemia models and a comprehensive evaluation of the pathophysiological changes.

### 2.4 Sample collection and testing

#### 2.4.1 Biochemical analysis

During the experimental phase, detailed daily records were maintained for each rat group, including water and food consumption, defecation and urination frequency, fur color, and behavior. Body weight was measured, and tail vein blood was collected on days 9, 18, and 27 to determine fasting SUA levels using an automatic biochemical analyzer.

On the 30th day, after a fasting period with access to water, urine samples were collected over 24 h using metabolic cages. Urine volume (UV) was recorded, followed by analyses of urine creatinine (UCr) and urine urea nitrogen (UUr) concentrations. The endogenous creatinine clearance rate (CCr) was calculated using the formula: CCr = (UCr × UV)/SCr.

On the 31st day, the rats were anesthetized, and blood samples were obtained from the abdominal aorta. These samples were subsequently used to assess serum biochemical parameters and perform metabolomics assays.

#### 2.4.2 Histopathology examination

Following blood sample collection, kidney tissues were immediately excised on an ice-cooled platform and weighed to calculate the organ coefficients. For histological preservation, the tissues were fixed in 4% paraformaldehyde. The preserved tissues were then embedded in paraffin, sectioned, and stained using Hematoxylin and Eosin (H&E) staining. The stained tissue sections were examined under a microscope.

### 2.5 Metabolomics analysis

#### 2.5.1 Metabolites extraction

To extract metabolites, 100 μL of plasma from each rat sample was mixed with 400 μL of pre-chilled 80% methanol in Eppendorf tubes. The mixture was incubated on ice for 5 min before being centrifuged at 15,000 g and 4°C for 20 min. A portion of the resulting supernatant was diluted with LC-MS grade water to achieve a final methanol concentration of 53%. This solution was subjected to a second round of centrifugation under the same conditions to collect the clear supernatant for subsequent LC-MS/MS analysis. To ensure analytical consistency and reliability, quality control (QC) samples were prepared by pooling 10 μL from each plasma sample.

#### 2.5.2 UHPLC-MS/MS analysis

Ultra-high-performance liquid chromatography coupled with tandem mass spectrometry (UHPLC-MS/MS) analysis was conducted at Novogene Co., Ltd. (Beijing, China). Chromatographic separation was achieved using a Hypersil Gold column (100 × 2.1 mm, 1.9 μm particle size) under a 12-min linear gradient at a flow rate of 0.2 mL/min. The mobile phase consisted of eluent A (0.1% formic acid in water) for positive polarity mode and eluent B (methanol) for negative polarity mode. The solvent gradient was set as follows: 2% B, 1.5 min; 2%–85% B, 3 min; 85%–100% B, 10 min; 100%–2% B, 10.1 min; 2% B, 12 min.

Mass spectrometric detection was conducted using the Q Exactive™ HF mass spectrometer, operating in both positive and negative ionization modes with a spray voltage set at 3.5 kV. The capillary temperature was regulated at 320°C. Sheath and auxiliary gas flows were maintained at 35 psi and 10 L/min, respectively, with the S-lens RF level set at 60. The auxiliary gas heater temperature was kept constant at 350°C.

#### 2.5.3 Data preprocessing and metabolite identification

The raw data from the UHPLC-MS/MS analyses were processed with Compound Discoverer 3.3 (CD3.3, Thermo Fisher Scientific) for peak alignment, peak detection, and metabolites quantification. The key parameters were configured as follows: peak areas were adjusted based on the first QC sample, with a mass tolerance of 5 ppm, a signal intensity tolerance of 30%, and a minimum intensity threshold.

The molecular formula of each metabolite was predicted using the normalized data, taking into account molecular ion peaks, additive ions, and fragment ions. Peak matching was conducted using databases such as mzVault, mzCloud, and MassList. Statistical analysis was performed using Python (version 2.7.6) and R (version 3.4.3) software for metabolite identification and relative quantification.

Metabolites with a relative standard deviation (RSD) in QC sample peak areas exceeding 30% were excluded. Metabolite annotation and comprehensive metabolic profiling were performed using databases such as KEGG (https://www.genome.jp/kegg/pathway.html), HMDB (https://hmdb.ca/metabolites) and LIPID MAPS (http://www.lipidmaps.org/).

#### 2.5.4 Data analysis

Data analysis was performed using multivariate statistical techniques such as principal component analysis (PCA) and orthogonal partial least squares-discriminant analysis (OPLS-DA) with SIMCA 14.1 software (Umetrics AB, Umeå, Sweden).

Permutation testing, conducted 200 times, was applied to the OPLS-DA models to prevent overfitting, ensuring the validity of the model’s predictive capability. Metabolites were then screened for significance based on Variable Importance in the Projection (VIP) scores (VIP > 1), fold-change (FC > 2 or < 0.5) values, and P-values (*p* < 0.05).

Hierarchical Clustering Analysis (HCA) was employed to analyze the distribution and clustering of the differentiated metabolites, with heatmaps generated with the R software. This analysis was based on the normalized relative quantities of these metabolites, aiming to elucidate systematic variations and similarities among the groups.

MetOrigin (http://metorigin.met-bioinformatics.cn/) was used for tracing the origin of differential metabolites. Origin analysis, functional analysis, and Sankey network analysis were all conducted using the simple MetOrigin analysis mode available on the official website.

Additionally, Receiver Operating Characteristic (ROC) curve analysis was used to determine the predictive accuracy of the metabolites. This was achieved using the “pROC” package in R, establishing a threshold to effectively discriminate between the different conditions reflected in the metabolic data.

### 2.6 Statistical analysis

Biochemical data were analyzed using SPSS 25.0 software (SPSS Inc., Chicago, IL, United States). One-way Analysis of Variance (ANOVA) was used to determine the differences among the three hyperuricemia rat models and the normal control group. Post hoc comparisons were conducted using the Least Significant Difference (LSD) method to identify specific group differences. T-tests were performed to assess differences between each model group and its corresponding positive control group. The relationships between differential metabolites and liver and kidney function parameters were examined through Pearson or Spearman correlation analyses, depending on the data distribution. All results are presented as mean ± standard deviation (SD), with significance level set at *p* < 0.05.

## 3 Results

### 3.1 Analysis of general conditions

#### 3.1.1 Condition of rats with hyperuricemia

During the experimental period, the control group rats displayed excellent health, as demonstrated by their energetic behavior and pristine fur condition. Conversely, rats in the hyperuricemia model groups exhibited signs of health deterioration, such as reduced activity levels, slower body weight gain, and deteriorating fur condition.

#### 3.1.2 Food and water intake, and body mass changes in hyperuricemia rats

Throughout the modeling phase, no mortality was observed in any of the hyperuricemia model or positive control groups. A substantial reduction in food consumption was noted across all hyperuricemia model groups compared to the NC group (*p* < 0.01), with the most significant decrease observed in the M2 group, where food intake was reduced by 37.8%–60.0% relative to the NC group (Figure 1B). Water intake in the M2 group fluctuated compared to the NC group, whereas the M3 group showed a marked increase in water consumption by the fourth week, reaching a significant peak (*p* < 0.01) (Figure 1C).

Regarding body mass, all hyperuricemia model groups showed a slower rate of weight gain compared to the NC group. Notably, the M3 group experienced the most significant weight reduction, with a 16% decrease relative to the NC group by the end of the study (Figure 1D). Between the three positive control groups and their corresponding model groups, no substantial differences were observed in food and water consumption or body weight.

### 3.2 Clinical chemistry analysis

#### 3.2.1 Dynamics of serum uric acid in hyperuricemic models

**FIGURE 2:**
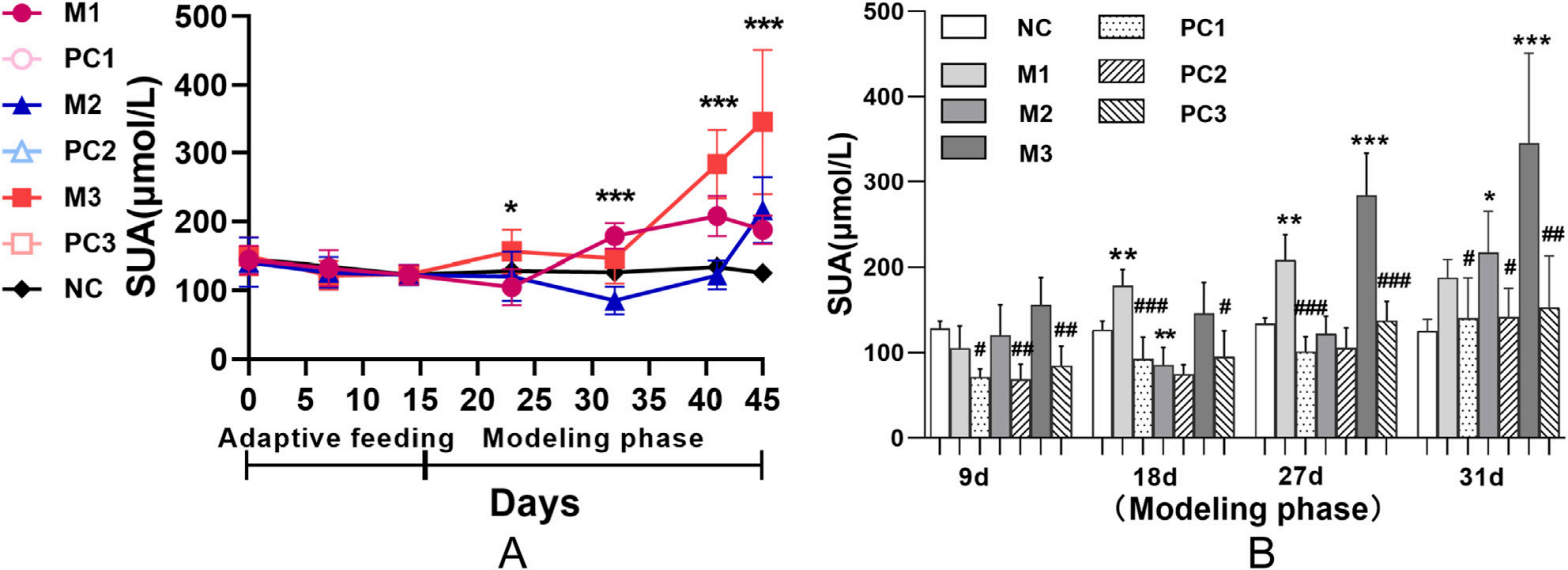
Serum uric acid dynamics in hyperuricemic rats using various induction protocols. Panel **(A)** shows group comparisons via ANOVA (^*^ *P* < 0.05, ^**^ *P* < 0.01, ^***^ *P* < 0.001). Panel **(B)** uses the LSD test for model-control group differences, and t-tests compare each model group with its positive control (^#^ *P* < 0.05, ^##^ *P* < 0.01, ^###^ *P* < 0.001). Data are means ± SD (n = 6).

During the adaptive feeding period, the SUA levels remained stable across all groups, showing no statistically significant differences (*p* > 0.05) and establishing a uniform baseline for the study (Figure 2A). At the start of the modeling phase, distinct temporal patterns in SUA levels emerged, corresponding to the three hyperuricemia induction methods. Compared to the NC group, each model group initially exhibited a decrease in SUA levels, followed by a significant rise (*P* < 0.05). Specifically, the M1 group displayed an early reduction in SUA levels, which then increased and stabilized. Conversely, the SUA levels in the M3 group increased rapidly and significantly. The M2 group’s SUA levels, however, showed a more gradual increase.

Figure 2B demonstrates that the positive control groups did not exhibit a significant increase in SUA levels compared to their respective model groups (*P* < 0.05), suggesting the potential for reversibility of hyperuricemia in these models. Moreover, this trend underscores the efficacy of allopurinol in attenuating the elevation of SUA levels under hyperuricemic conditions.

#### 3.2.2 Renal function implications of hyperuricemia models

**FIGURE 3:**
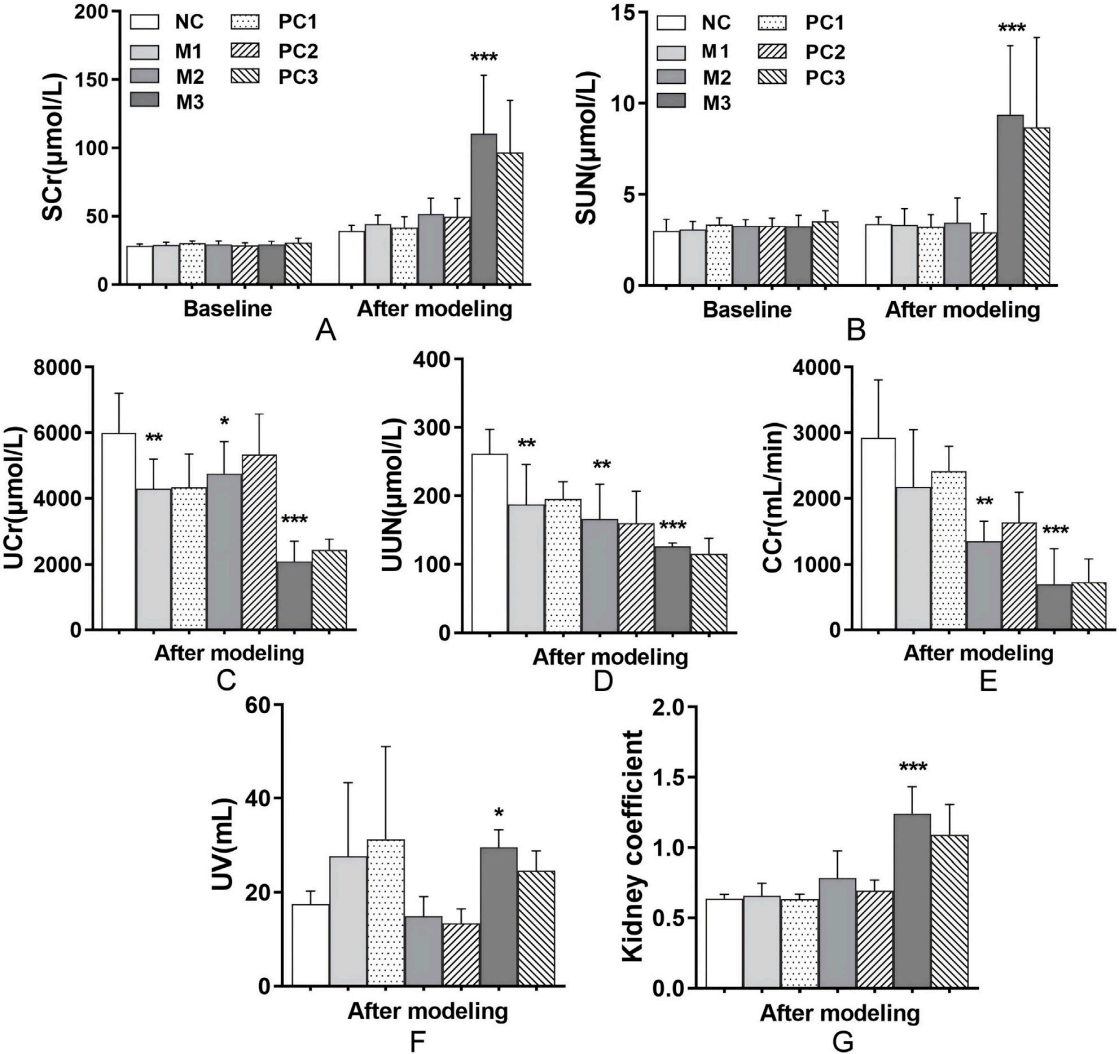
Renal function alterations under different hyperuricemia induction methods. This figure presents the effects of different hyperuricemia induction methods on renal function, evaluated through markers such as **(A)** SCr, **(B)** SUN, **(C)** UCr, **(D)** UUN, **(E)** CCr, **(F)** UV, and **(G)** kidney coefficients. Data are means ± SD (n = 6). Statistical significance for the variations between the model groups and the normal control group is indicated by ^*^ *P* < 0.05, ^**^ *P* < 0.01, and ^***^ *P* < 0.001. Differences between the model groups and their respective positive control groups are denoted by ^#^ *P* < 0.05, ^##^ *P* < 0.01, and ^###^ *P* < 0.001.

Serum creatinine (SCr) and serum urea nitrogen (SUN) are crucial clinical indicators for assessing renal function. Prior to hyperuricemia development, baseline levels of SCr and SUN remained consistent across all groups, establishing a uniform foundation for the study conditions (*P* > 0.05, Figures 3A,B). Following the modeling phase, no significant alterations in SCr and SUN levels were observed in the M1 and M2 groups relative to the NC group (*P* > 0.05). In contrast, the M3 group demonstrated a marked increase in both SCr and SUN levels (*P* < 0.001), signaling considerable renal impairment. The positive control groups did not show significant improvement in these biomarkers compared to the elevated levels in the M3 group (*P* > 0.05).

Compared to the control, all hyperuricemia models exhibited significant reductions in urine creatinine (UCr), urine urea nitrogen (UUN), and creatinine clearance rate (CCr) (*P* < 0.05, Figures 3C–E), indicating impaired uric acid excretion efficiency. Notably, the M3 group showed pronounced increases in urine volume (UV) and kidney coefficients (*P* < 0.05 and *P* < 0.001, respectively, Figures 3F,G), which suggests severe renal dysfunction. These results highlight a broad decline in uric acid clearance across the models, with the M3 group showing the most significant impact. Despite these alterations, the positive control groups did not exhibit a significant improvement in renal function compared to their respective model groups (*P* > 0.05, Figures 3C–E).

#### 3.2.3 Impact of hyperuricemia models on liver function

**FIGURE 4:**
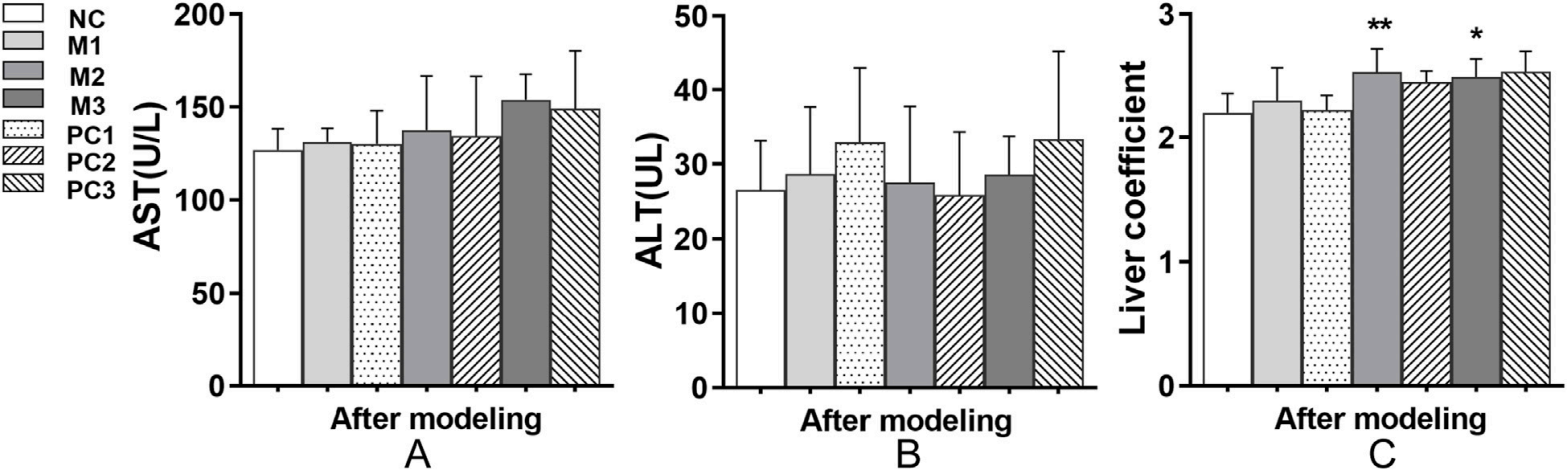
Liver function alterations under different hyperuricemia induction protocols. **(A)** AST levels, **(B)** ALT levels, and **(C)** liver coefficients are shown. Data are means ± SD (n = 6). Statistical significance of the differences between the model groups and the normal control group is denoted by ^*^ *P* < 0.05, ^**^ *P* < 0.01, and ^***^ *P* < 0.001. Variations between the model groups and their respective positive control groups are represented by ^#^ *P* < 0.05, ^##^ *P* < 0.01, and ^###^ *P* < 0.001.

Aspartate aminotransferase (AST) and alanine aminotransferase (ALT) are crucial biomarkers for liver function assessment in clinical diagnostics. Following the modeling phase, AST and ALT levels in the hyperuricemia model groups remained comparable to those in the NC group, with no significant differences observed (*P* > 0.05, Figures 4A,B). However, the liver coefficients in the M2 and M3 groups showed significant increases relative to the NC group (*P* < 0.05, Figure 4C), indicating potential hepatic alterations, as evidenced by changes in liver size or mass. Additionally, the positive control groups did not exhibit a significant decrease in liver coefficients compared to their corresponding model groups (*P* > 0.05, Figure 4C).

### 3.3 Histological evaluation

**FIGURE 5:**
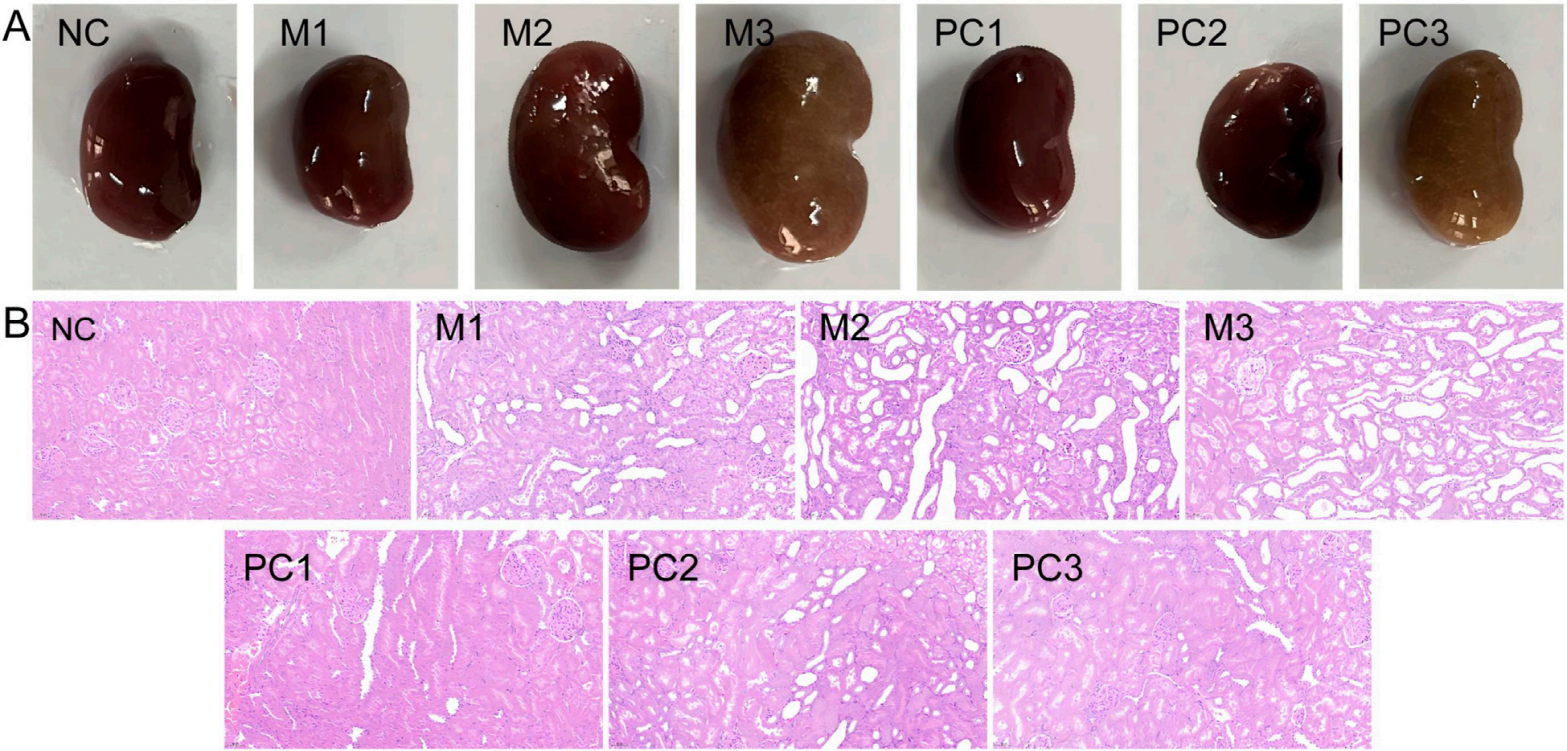
Comparative analysis of renal gross anatomy **(A)** and histopathology with H&E staining [**(B)**, ×200] in various groups.

In Figure 5A, the gross observation of the kidneys shows that in the NC group, the kidneys are dark red, with smooth and glossy surfaces and no swelling. In contrast, the kidneys in all three model groups are enlarged, especially in the M3 group, where the kidneys display distinct crystalline granules on their surfaces and are dark yellow. In the positive control groups, PC1 and PC2, a significant reduction in kidney enlargement is observed. The H&E staining results (Figure 5B) reveal that the kidney tissue in the NC group maintains a mostly normal structure, with uniform thickness of the renal tubular walls and broadly normal glomerular architecture. However, the kidneys in the three model groups exhibit varying degrees of thinning and atrophy of the renal tubular walls, detachment of epithelial cells and brush borders, and dilation of renal tubules. The M3 group is characterized by severe inflammatory cell infiltration around the renal tubules and marked cytoplasmic vacuolar degeneration in epithelial cells, indicating the most severe damage to renal tissue in this group. Compared to the model groups, the positive control groups show some pathological improvement.

### 3.4 Plasma metabolomics profiling

#### 3.4.1 Multivariate data analysis

**FIGURE 6:**
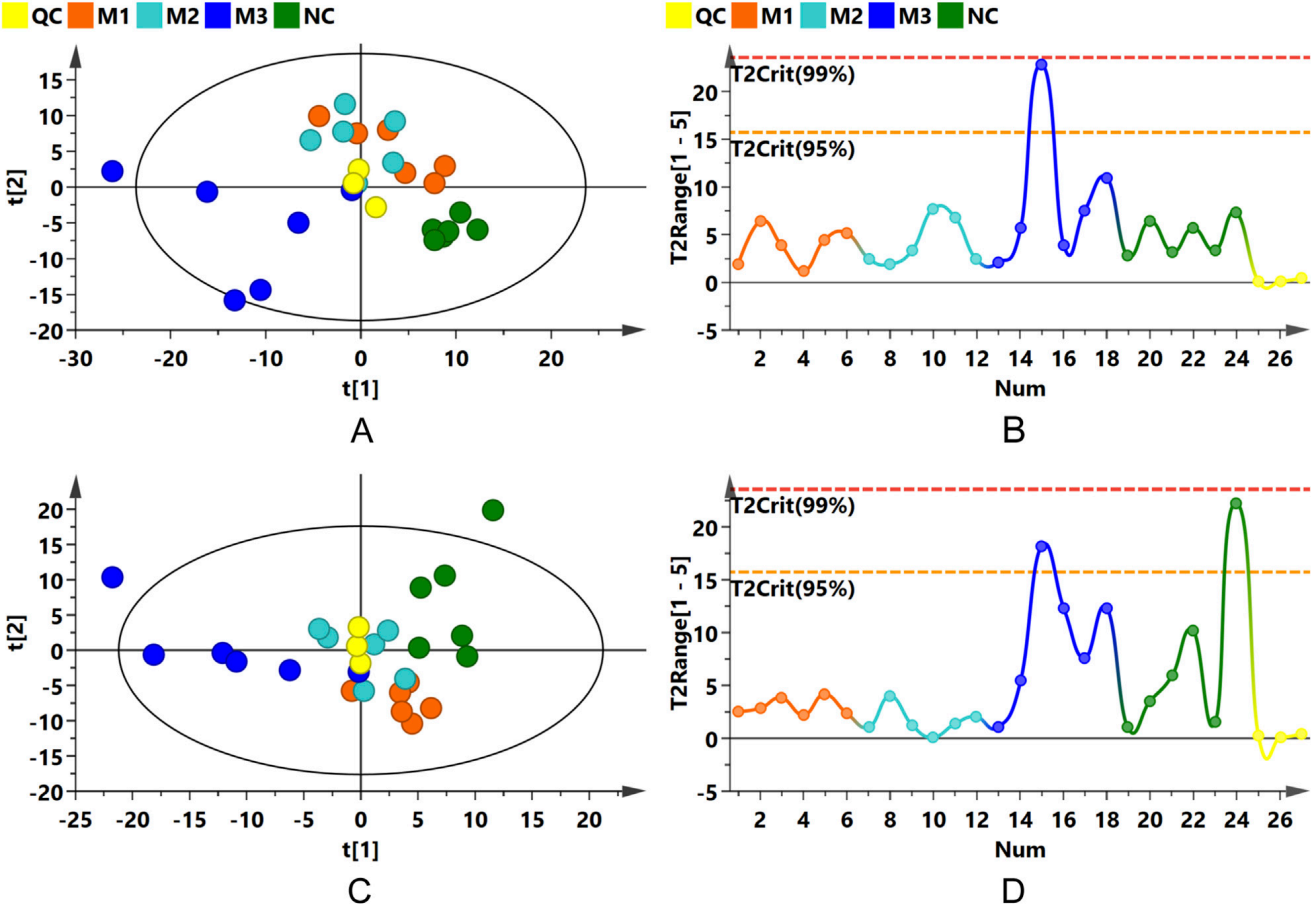
PCA and Hotelling’s T2 Plots. **(A,B)** Present PCA score and Hotelling’s T2 plots for the positive ion mode; **(C,D)** for the negative ion mode.

PCA, an unsupervised statistical method, was applied to assess the overall metabolic variances and to pinpoint outliers within the dataset. The PCA score plots for both ionization modes, as illustrated in Figures 6A,C, exhibited distinct separations between the model groups and the NC group. This differentiation suggests notable variances in the serum endogenous metabolite profiles. QC samples clustered tightly near the PCA plot origin, affirming the consistency of the analytical method (Figures 6A,C). Additionally, Hotelling’s T2 plot, used to identify strong outliers (indicated by surpassing the red dashed line), revealed no significant outliers, corroborating the serum metabolomics analysis system’s robustness, stability, and reproducibility (Figures 6B,D).

OPLS-DA was subsequently conducted to confirm the differences observed in the PCA, with score plots displaying clustering patterns among samples from the various groups in both positive and negative ion modes. Although some overlap was observed between the M1 and M2 groups, distinct clustering away from the NC group was evident for all model groups (Supplementary Figures S1A,C). Permutation tests performed on the OPLS-DA models confirmed their reliability and stability (Supplementary Figures S1B,D).

**FIGURE 7:**
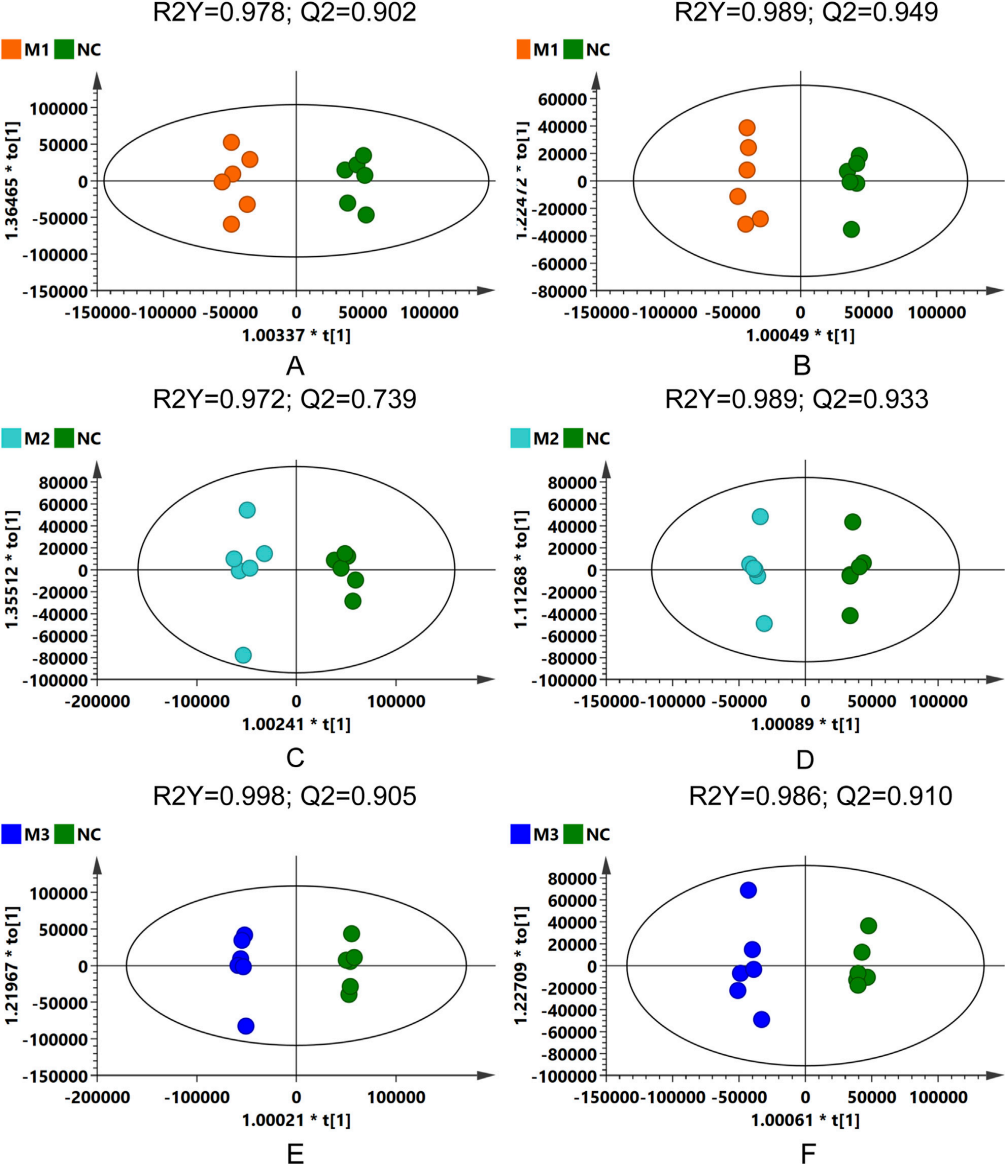
OPLS-DA score plots: comparative analysis across model groups and NC group in different ionization modes. **(A,C,E)** for positive ion mode, and **(B,D,F)** for negative ion mode.

The distinct separations among the three model groups and the control group were further substantiated in the OPLS-DA score plots (Figure 7), with permutation test outcomes (Supplementary Figure S2) indicating the absence of model overfitting. These analyses suggest that the induction methods for hyperuricemia resulted in distinct serum metabolic alterations in the rats, establishing a reliable model for identifying differential metabolites between the model and NC groups.

#### 3.4.2 Differential metabolites screening

In this analysis, 410 metabolites were detected in positive ion mode and 283 in negative ion mode. Employing VIP scores, *P* values, and FC as criteria for analysis, 205 metabolites were determined to be significantly altered in the comparison pairs M1-NC, M2-NC, and M3-NC. Supplementary Figure S3A presents a clustering heatmap of these differential metabolites, with pronounced clustering observed between the M1 and M2 groups indicating similarity in their metabolic expression profiles and pathways, distinct from the M3 group, highlighting the unique metabolic alterations triggered by the various hyperuricemia induction models.

**FIGURE 8:**
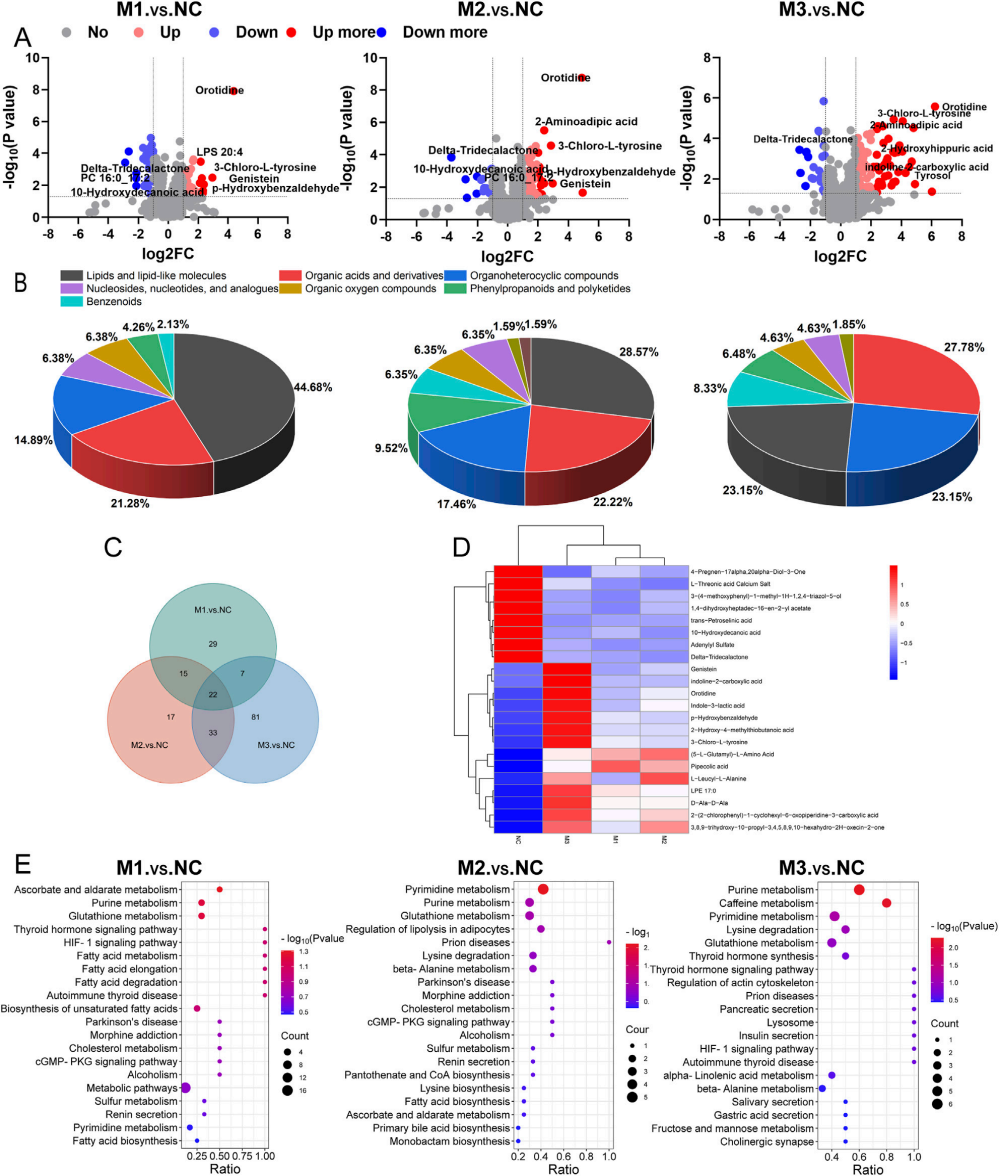
Plasma metabolomics profiling. **(A)** Volcano plots for differential metabolites; **(B)** Pie charts categorizing metabolites; **(C)** Venn diagram showing the intersection of differential metabolites among the three comparison groups (M1-NC, M2-NC, M3-NC); **(D)** Heatmap of intersecting differential metabolites; **(E)** KEGG pathway mapping across comparison groups.

Volcano plots (Figure 8A) illustrated the distribution of differential metabolites between the model groups and the NC group. Specifically, 73 metabolites differentiated the M1 group from the NC, with 35 being upregulated and 38 downregulated. For the M2 group compared to the NC, 87 metabolites were identified as differential, with 70 upregulated and 17 downregulated. The M3 group exhibited the most pronounced variation, with 143 differential metabolites, of which 112 were upregulated and 31 downregulated. As shown in Figure 8B, the predominant classes of these differential metabolites—lipids and lipid-like molecules, organic acids and their derivatives, and organoheterocyclic compounds—comprised approximately 75% of the total identified differential metabolites, highlighting the significant impact of hyperuricemia on these metabolic categories. Heatmap analysis of the differential metabolites within the M1-NC, M2-NC, and M3-NC comparisons (Supplementary Figure S3B) illustrated their expression trends, showing distinct clustering patterns within each group and significant variations between groups.

In Figure 8C, a Venn diagram identifies 22 metabolites shared across the three comparisons, highlighting potential biomarkers for hyperuricemia. These are predominantly amino acids and lipids, with 14 metabolites upregulated and 8 downregulated, as detailed in Supplementary Table S1. The clustering heatmap (Figure 8D) further demonstrated significant changes and a consistent trend in these 22 metabolites across the models compared to the NC group. Notably, the M3 group exhibited the most pronounced changes in metabolite levels, indicating severe metabolic disruptions.

#### 3.4.3 KEGG pathway analysis

The metabolic pathway analysis involved mapping significant differential metabolites identified across the M1-NC, M2-NC, and M3-NC comparison groups to the KEGG pathway database, as depicted in Figure 8E. In the M1-NC comparison, significant alterations were observed in pathways related to ascorbate and aldarate metabolism, purine metabolism, and glutathione metabolism. Key findings included elevated levels of L-ascorbate, adenosine, inosine, and (5-L-glutamyl)-L-amino acids, along with a reduction in dehydroascorbic acid and adenylyl sulfate levels. For the M2-NC group, notable impacts were seen on pyrimidine metabolism, purine metabolism, and glutathione metabolism. This was characterized by increased concentrations of metabolites such as uracil, pseudouridine, orotidine, 5-methylcytosine, ureidosuccinic acid, adenosine, inosine, and (5-L-glutamyl)-L-amino acids, and decreased levels of adenylyl sulfate, spermine, and dehydroascorbic acid. In the M3-NC comparison, pathways including purine metabolism, caffeine metabolism, pyrimidine metabolism, lysine degradation, and glutathione metabolism were significantly affected, showing elevated levels of metabolites such as uric acid, 1-methyluric acid, uracil, pseudouridine, orotidine, 5-methylcytosine, ureidosuccinic acid, 2-aminoadipic acid, 5-aminopentanoate, pipecolic acid, (5-L-glutamyl)-L-amino acids, and L-ascorbate, with reductions in adenylyl sulfate, xanthosine, deoxyinosine, 2,6-dihydroxypurine, xanthine, L-glutathione oxidized, and spermine.

**FIGURE 9:**
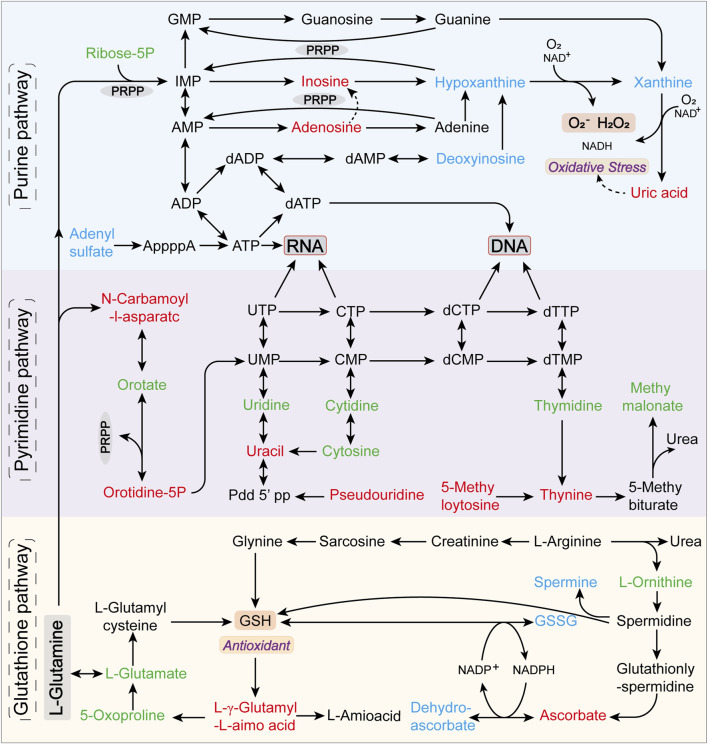
Predominantly disrupted metabolic pathways and their interrelations among metabolites in hyperuricemic rats. Metabolites annotated in green are identified but not significantly altered, red indicates upregulated differential metabolites, and blue denotes downregulated differential metabolites.

Notably, the purine metabolism, pyrimidine metabolism, and glutathione metabolic pathways were significantly altered in at least two models, underscoring the key metabolic pathways most disrupted during hyperuricemia onset. Figure 9 illustrates the critical metabolic pathways and the biochemical interconnections among metabolites in hyperuricemic rats, revealing the pivotal role of oxidative stress in the progression of hyperuricemia.

#### 3.4.4 Metorigin tracing analysis of differential metabolites

**FIGURE 10:**
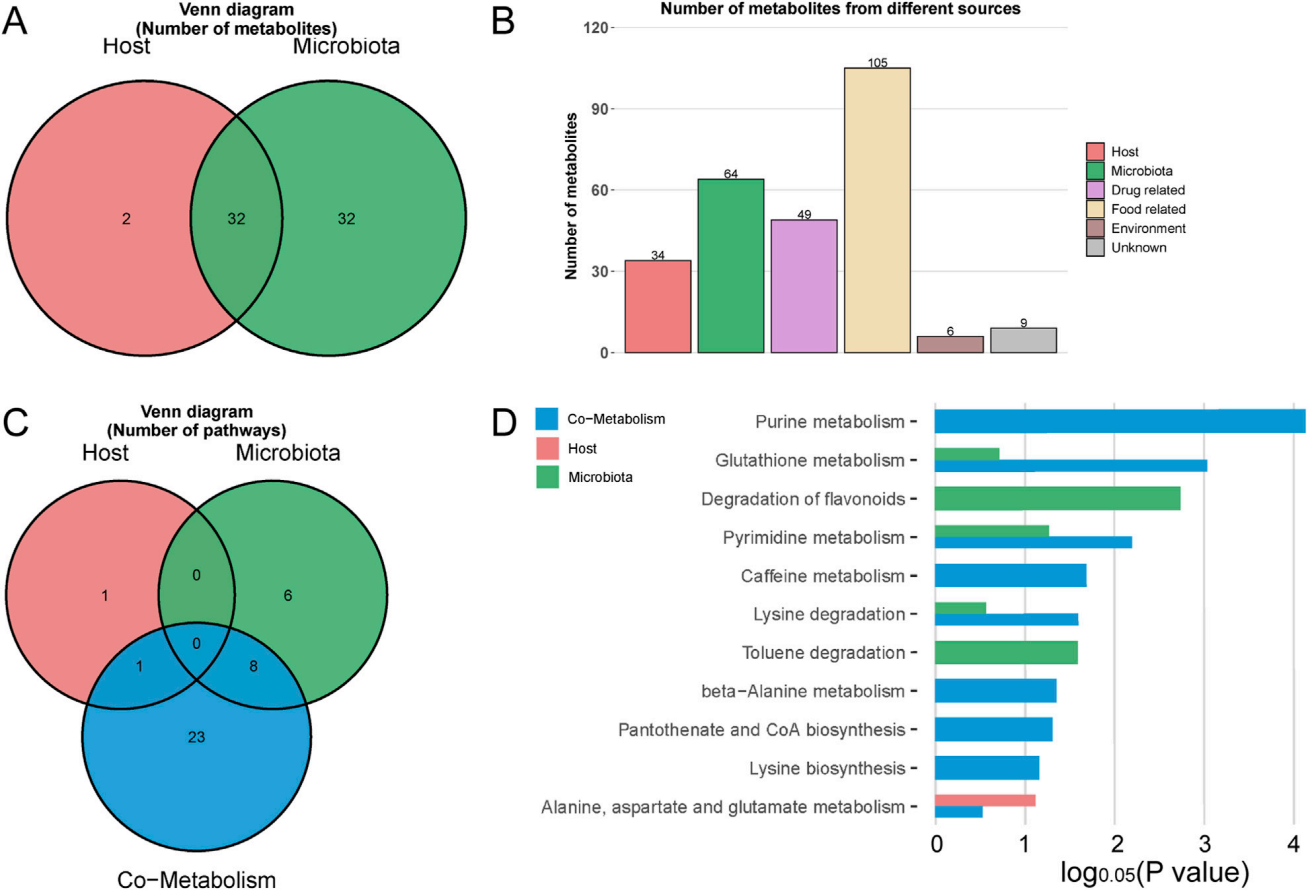
MetOrigin tracing analysis of differential metabolites. **(A,B)** represent the Venn diagram and histogram of tracing differential metabolites, respectively; **(C,D)** depict the Venn diagram and histogram of enrichment analysis of differential metabolic pathways, respectively.

In a comparative analysis of the M1-NC, M2-NC, and M3-NC groups, a total of 204 differential metabolites associated with hyperuricemia were identified (Figure 8C), 119 of which corresponded with entries in the KEGG and HMDB databases. MetOrigin tracing analysis was applied to these 119 metabolites to investigate the interactions between the gut microbiome and metabolome. This analysis identified 32 microbiota-host co-metabolites, 2 host-specific metabolites, and 32 microbiota-specific metabolites, as illustrated in Figures 10A,B. Functional analysis showed that 1, 6, and 32 metabolic pathways corresponded with host, microbiota, and co-metabolism databases, respectively (Figure 10C). Figure 10D highlights significant metabolic pathways associated with hyperuricemia (log_0.05_ *P* value > 1), where, apart from the degradation of flavonoids and toluene degradation pathways that are microbiota-specific, the remaining primarily involve host-microbiota co-metabolism. Key among these are purine metabolism, glutathione metabolism, and pyrimidine metabolism, which are the most critical co-metabolic pathways related to hyperuricemia (log_0.05_ *P* value > 2), thereby validating the KEGG pathway enrichment results.

Additionally, Sankey network visualization was utilized to delineate the statistical correlations and biological relationships between microbial communities and metabolites. Specifically, within the purine metabolism pathway, multiple metabolic reactions (R01768, R01769, R02103, and R02107) were found to be instrumental in the critical pathway converting hypoxanthine and xanthine into uric acid. The phyla *Pseudomonadota*, *Actinomycetota*, and *Ascomycota* were identified as the primary gut phyla intimately linked to these reactions (Supplementary Figure S4).

#### 3.4.5 Association between plasma metabolites and hyperuricemia

Metabolites present in at least two of the model groups and enriched in key metabolic pathways were subjected to classical univariate ROC analysis. This analysis aimed to identify potential biomarkers for hyperuricemia, with the detailed findings presented in Table 1. Eleven metabolites, each exhibiting an AUC greater than 0.90, were identified. Notably, orotidine, (5-L-glutamyl)-L-amino acids, and adenylyl sulfate were recognized as differential metabolites across all three model groups, underscoring their critical roles in hyperuricemia.

**TABLE 1:** ROC analysis of metabolites for hyperuricemia prediction.

Metabolite	AUC			Label	Pathway involved
	M1 vs. NC	M2 vs. NC	M3 vs. NC		
Uracil	-	1.00	1.00	up	Pyrimidine metabolism
Pseudouridine	-	0.97	1.00	up	Pyrimidine metabolism
Orotidine	1.00	1.00	1.00	up	Pyrimidine metabolism
5-Methylcytosine	-	1.00	1.00	up	Pyrimidine metabolism
Ureidosuccinic acid	-	1.00	1.00	up	Pyrimidine metabolism
Adenylyl Sulfate	1.00	1.00	0.97	down	Purine metabolism
Adenosine	1.00	0.94	-	up	Purine metabolism
Inosine	0.97	0.94	-	up	Purine metabolism
Spermine	-	0.94	1.00	down	Glutathione metabolism
(5-L-Glutamyl)-L-Amino Acid	1.00	1.00	1.00	up	Glutathione metabolism
Dehydroascorbic acid	0.97	0.94	-	down	Glutathione metabolism
L-Ascorbate	0.97	-	1.00	up	Glutathione metabolism

**FIGURE 11:**
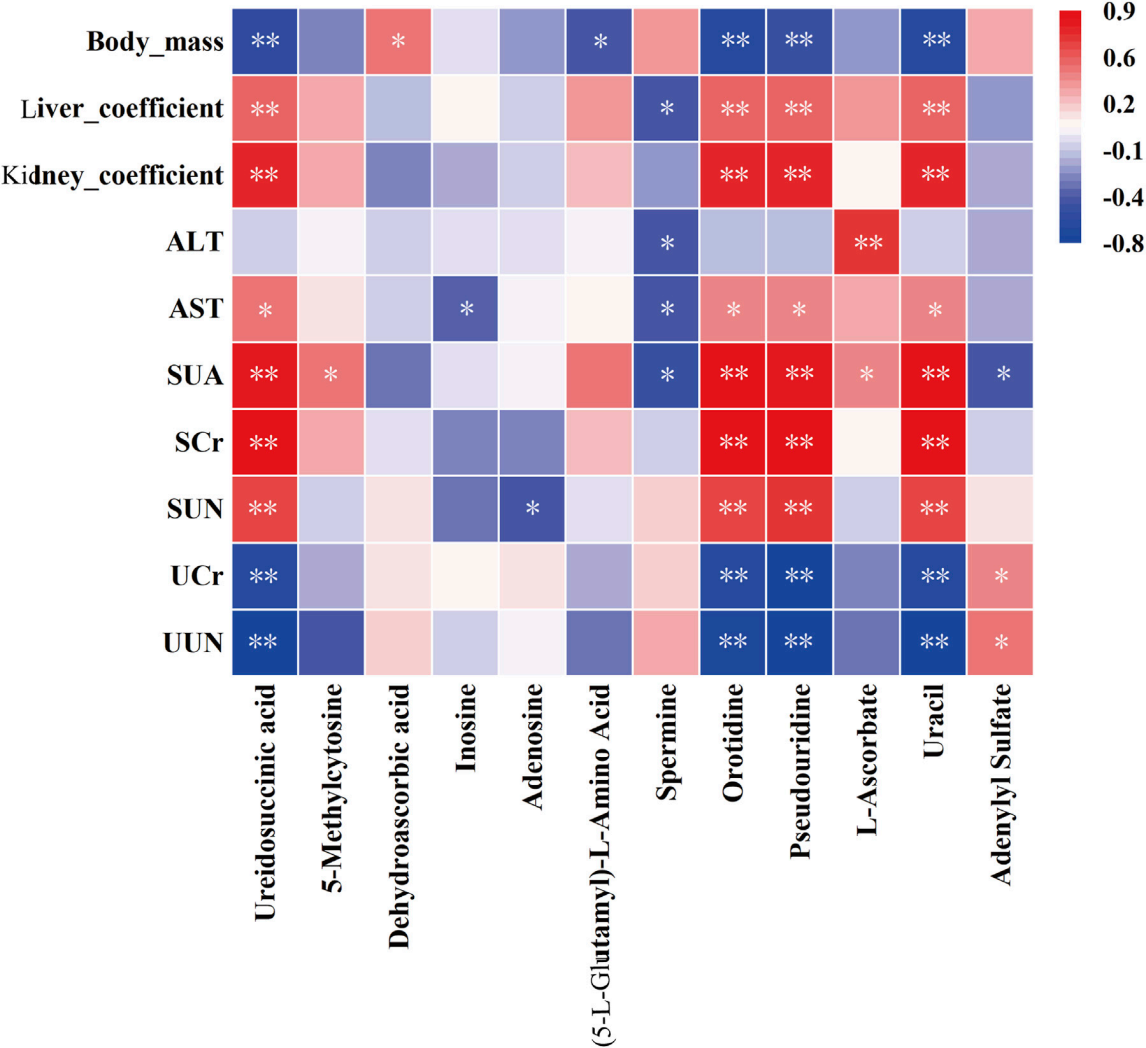
Correlation analysis between plasma metabolites and biochemical indices. Statistical significance denoted as ^*^ *P* < 0.05, ^**^ *P* < 0.01.

Spearman’s correlation analysis was conducted to evaluate the relationships between selected metabolites and liver/kidney function indicators, with the results presented in Figure 11. This analysis revealed significant and consistent correlations for orotidine, ureidosuccinic acid, uracil, and pseudouridine with liver and kidney function markers. These metabolites were established as optimal discriminators for hyperuricemia, demonstrating high predictive accuracy with AUC values of 0.97 or higher.

## 4 Discussion

Hyperuricemia, ranked alongside hypertension, hyperglycemia, and hyperlipidemia as a major health concern, is closely associated with a spectrum of metabolic disorders (Yanai et al., 2021; Johnson et al., 2018) and serves as an independent risk factor for hyperuricemic nephropathy (Sharaf El Din et al., 2017; Niu et al., 2022; Li et al., 2022). The development of stable and reliable animal models is crucial for the advancement of hyperuricemia research and the discovery of novel therapeutic agents. In rodents, the natural uricase activity that converts uric acid into allantoin (Lu et al., 2019) highlights the importance of uricase inhibition in creating effective disease models. Accordingly, our study utilized potassium oxonate, a competitive uricase inhibitor, both alone and in combination with fructose and adenine, to successfully elevate uric acid levels in rats, thereby establishing and validating robust hyperuricemia models.

During the modeling phase, we observed a consistent pattern in which SUA levels initially decreased and then significantly increased across all methods employed. This fluctuation is likely due to a transient increase in uric acid following the administration of potassium oxonate, potentially enhancing uricase activity or expression to maintain uric acid homeostasis, leading to a subsequent decrease in uric acid levels (Wen et al., 2020). However, with prolonged exposure, a compensatory imbalance became evident, marked by the cumulative effect of potassium oxonate, which resulted in continuous uricase inhibition and elevated blood uric acid levels.

The three modeling methods exhibited distinct temporal patterns in SUA levels. The M3 group showed the earliest and most significant rise in SUA, initially decreasing due to uricase feedback before consistently rising. Interestingly, the M2 group, which combined 20% fructose water with potassium oxonate, did not achieve higher SUA levels than the M1 group that used only potassium oxonate. This finding challenges the perceived benefits of combined modeling and also increases the cost of animal experiments, which contrasts with previous research that has supported the use of potassium oxonate and fructose for establishing a stable, long-term hyperuricemia model (Zhu et al., 2021). The difference might be attributed to variations in sample collection times: in our study, blood samples were collected the following morning after a 12-h fast without water restriction, unlike other studies that sampled blood 1 hour post the final potassium oxonate dose (Zhu et al., 2021). Furthermore, the positive control groups in each model exhibited reduced SUA levels compared to their respective model groups, indicating the efficacy of all three modeling approaches in evaluating uric acid-lowering drugs (Bao et al., 2022; Meng et al., 2021).

The kidney, essential for uric acid excretion, often incurs significant damage in cases of hyperuricemia (Niu et al., 2022; Wu et al., 2021). Research has established a definitive link between hyperuricemia and hepatic steatosis (Wan et al., 2016), or fatty liver (Xu, 2016), demonstrating the significant impact of elevated uric acid levels on hepatic lipid metabolism and overall metabolic balance (Yang et al., 2022). In our study, biochemical indices and pathological analyses revealed that the M1 model showed minimal kidney damage, whereas the M2 model caused early, mild kidney dysfunction. The M3 model, however, resulted in severe renal impairment. The methodologies employed in our research effectively induced varying degrees of renal damage, addressing diverse research and clinical needs in hyperuricemia. This variation in organ impairment allows for the selection of models tailored to specific experimental or therapeutic goals, thereby enhancing the relevance and application of our findings in hyperuricemia research. The positive control groups did not show significant improvements in kidney function compared to the model groups, highlighting the need to focus on both uric acid reduction and organ health in hyperuricemia management. Notably, none of the modeling methods significantly affected serum AST and ALT levels, suggesting that the duration of the modeling period may have influenced these results (Zhu et al., 2021). This observation underscores the importance of considering both the modeling duration and the comprehensive effects of therapeutic agents on kidney functions in the development and evaluation of hyperuricemia models.

The regulation of uric acid homeostasis critically depends on the balance between synthesis and excretion. Recent studies indicate that disruptions in the excretory mechanisms of uric acid, primarily through renal and intestinal pathways, are responsible for more than 90% of hyperuricemia cases in clinical settings (Terkeltaub et al., 2006). Our study, employing three distinct methodologies to establish rat models of hyperuricemia, revealed a significant reduction in urinary excretion of uric acid and urea nitrogen compared with the control group. This finding suggests potential obstacles in the uric acid elimination process (Perez-Ruiz et al., 2002), potentially related to abnormalities in uric acid transport protein expression (Mandal and Mount, 2015; So and Thorens, 2010; Nakayama et al., 2017). The precise mechanisms behind these excretory challenges, however, require further investigation.

Moreover, our study conducted a comprehensive assessment of serum metabolic changes in hyperuricemic rats via non-targeted metabolomics. This analysis identified significant alterations in the metabolism of purine, pyrimidines, and glutathione, which are crucially linked to the development of hyperuricemia. These metabolic shifts highlight the complex interplay between uric acid production, excretion, and oxidative stress, underscoring the multifaceted nature of this disorder. Additionally, metabolite tracing analysis utilizing MetOrigin indicated that the metabolites associated with hyperuricemia predominantly originate from microbial sources, with the metabolic pathways largely involving host-microbiome co-metabolism. This finding underscores the significant influence of the gut microbiome on the pathogenesis of hyperuricemia.

Purines and their derivatives, particularly adenosine and adenosine triphosphate (ATP), play fundamental roles in regulating intracellular energy balance and nucleotide synthesis (Huang et al., 2021). This study revealed that abnormalities in purine metabolism, such as elevated levels of adenosine and inosine, along with reduced adenylyl sulfate, are critical for identifying metabolic imbalances underlying hyperuricemia and pinpointing potential therapeutic targets. Adenosine deaminase (ADA) facilitates the conversion of adenosine to inosine, both of which serve as precursors to uric acid. Elevated levels of these compounds suggest either accelerated purine metabolism or dysfunction in the uric acid excretion mechanism, leading to increased SUA concentrations. Recent research has focused on new drug developments targeting these metabolic pathways (Du et al., 2023; Wang et al., 2019; Lin et al., 2022). For instance, studies by Wang et al. demonstrated that *Lactobacillus DM9218* significantly degrades inosine in the gut, reducing uric acid production induced by a high-fructose diet and decreasing inosine circulation in the liver (Wang et al., 2019). This highlights the potential of inosine as a biodegradable therapeutic target for treating hyperuricemia and its renal complications. Furthermore, explorations using the Bio-Sankey network revealed that bacterial phyla such as *Pseudomonadota*, *Actinomycetota*, and *Ascomycota* are closely related to the key processes of uric acid production from hypoxanthine and xanthine in purine metabolism. Research has indicated that hypoxanthine and xanthine, when produced by bacteria, contribute to the regeneration of the intestinal mucosal barrier and protection of intestinal integrity (Lee et al., 2020). Currently, the gut microbiome is recognized as a novel target for managing hyperuricemia. Research has shown that the gut microbiota, through the secretion of active enzymes, participates in the breakdown and metabolism of purines and uric acid, with genera such as *Lactobacillus and Pseudomonadota* synthesizing urate oxidase to enhance uric acid degradation and ultimately facilitate urea excretion (Wang et al., 2022). Li et al. highlighted the role of three ribonucleoside hydrolases—RihA, B, and C in *Lactiplantibacillus plantarum*—in catalyzing the conversion of nucleosides into bases, thereby aiding in the regulation of urate metabolism in mice on a high-nucleoside diet (Li et al., 2023). Moreover, recent studies by Kasahara et al. and Liu et al. have further advanced the understanding of urate degradation by the gut microbiome, proposing that microbial purine degradation serves as a crucial regulatory mechanism in maintaining purine homeostasis and stabilizing circulating uric acid levels within the host (Liu et al., 2023; Kasahara et al., 2023).

Purine and pyrimidine metabolism are intricately interconnected within biological systems, playing essential roles in the synthesis and breakdown of nucleic acids, and providing the necessary nucleotides for DNA and RNA production (Edwards and Fox, 1984). Phosphoribosyl pyrophosphate synthetase (PRPP synthetase), a pivotal enzyme in both metabolic pathways (Dewulf et al., 2022; Yang et al., 2020), is regulated by nucleotide and nucleoside levels, indicating a complex cross-regulation between purine and pyrimidine metabolism. In conditions such as hyperuricemia, an upsurge in purine metabolism and PRPP synthetase activity necessitates increased synthesis of pyrimidine nucleotides to balance the elevated purine nucleotides (Nyhan, 2005), thereby meeting the demand for DNA and RNA synthesis. Additionally, the accumulation of uric acid in the body may influence the pyrimidine metabolic pathway through a negative feedback mechanism (Ames et al., 1981), as evidenced by the increased serum levels of pyrimidine metabolites observed in this study, such as uracil, pseudouridine, orotidine, 5-methylcytosine, and ureidosuccinic acid.

Initial research has underscored the role of uric acid as a pivotal endogenous antioxidant, neutralizing singlet oxygen and free radicals, thus protecting enzymes like superoxide dismutase and alleviating oxidative stress caused by increased levels of reactive oxygen species (ROS) (Fabbrini et al., 2014; Zinellu and Mangoni, 2023). However, recent studies have highlighted the limitations of the antioxidant properties of uric acid, indicating that levels exceeding physiological norms can disrupt redox balance and intensify oxidative damage (Mandal and Mount, 2015; Ferreira et al., 2023). In the context of glutathione (GSH) metabolism, which involves synthesis from glutamate, cysteine, and glycine through the γ-glutamyl cycle and serves as a key intracellular antioxidant (Raghu et al., 2021), findings suggest that hyperuricemia induces metabolic disturbances. This is evidenced by elevated levels of (5-L-glutamyl)-L-amino acids and reduced levels of spermine. Such disturbances are likely due to increased production of ROS associated with elevated uric acid levels, leading to accelerated metabolism of GSH or enhanced activity of glutathione synthetase. Given the importance of spermine in cellular growth, differentiation, and DNA stability (Wang et al., 2023; Zhao et al., 2023), disturbances in the glutathione pathway in hyperuricemia may impair cellular defenses against oxidative stress, consequently increasing the risk of cellular damage.

Correlation analysis in our study revealed a significant association between elevated levels of pyrimidine metabolites, such as uracil, ureidosuccinic acid, orotidine, and pseudouridine, and organ damage in conditions of hyperuricemia. This damage was particularly evident in the kidneys. The analysis indicates the potential of these metabolites as powerful biomarkers for hyperuricemia, with a high predictive value (AUC ≥ 0.97). The kidneys, which are crucial for waste metabolism and elimination, experience stress due to the accumulation of these metabolites, leading to cellular and functional impairments (Cao et al., 2005). Specifically, elevated levels of uracil and ureidosuccinic acid may disrupt uric acid excretion, increasing renal stress and potentially leading to kidney stones or impaired renal function (Mazzali et al., 2001; Fathallah-Shaykh and Cramer, 2014). Furthermore, increased levels of orotidine and pseudouridine could signal enhanced RNA degradation and cellular damage (Kim et al., 2017; Li et al., 2016), particularly in the context of organ dysfunction (Simmonds et al., 1991). Dysregulation of pyrimidine metabolism may induce oxidative stress (Xu et al., 2022), resulting in cellular and tissue oxidative damage, characterized by the overproduction of free radicals or reactive oxygen species, thereby accelerating aging and triggering inflammation harmful to kidney health. Thus, monitoring these pyrimidine metabolites provides crucial diagnostic and prognostic insights for managing hyperuricemia and its related organ complications.

## 5 Conclusion

In this study, we established rat models of hyperuricemia with varying degrees of kidney damage by administering potassium oxonate both alone and in combination with fructose and adenine. The reversibility of these models was also confirmed. Serum metabolomics analysis revealed significant alterations in the metabolism of purine, pyrimidines, and glutathione, highlighting the critical role played by oxidative stress in this pathology. Notably, pyrimidine metabolites such as orotidine, ureidosuccinic acid, uracil, and pseudouridine, which are closely associated with liver and kidney damage, were identified as important and potential biomarkers for hyperuricemia. Furthermore, metabolite tracing analysis utilizing MetOrigin demonstrated that the gut microbiome significantly influences the pathogenesis of hyperuricemia. Future research should be directed towards elucidating the underlying mechanisms of these metabolic changes and developing novel therapies that effectively lower uric acid levels while protecting liver and kidney function. It is recommended that special focus be placed on the interactions between drug formulations, the gut microbiome, and hyperuricemia.

## Data Availability

The raw data supporting the conclusions of this article will be made available by the authors, without undue reservation.

## Supplementary material

Supplementary Figure S1 OPLS-DA score plots and permutation test results. **(A,B)** Show OPLS-DA score plots and permutation test results for positive ion mode; **(C,D)** for negative ion mode.

Supplementary Figure S2 Permutation test results: comparative analysis across model groups and NC group in different ionization modes. **(A,C,E)** for positive ion mode, and **(B,D,F)** for negative ion mode.

Supplementary Figure S3 Clustering heatmaps. **(A)** Global clustering heatmap of differential metabolites across hyperuricemia rat models (M1, M2, M3) and the NC group; **(B)** Pairwise clustering heatmaps comparing differential metabolites between each hyperuricemia model and the NC group.
