# Supplementary figures and images for: Evaluating renal injury characteristics in different rat models of hyperuricemia and elucidating pathological molecular mechanisms via serum metabolomics
Source: Front Pharmacol. 2024 Sep 2;15:1433991. doi: 10.3389/fphar.2024.1433991 (PMC11403331; doi:10.3389/fphar.2024.1433991)

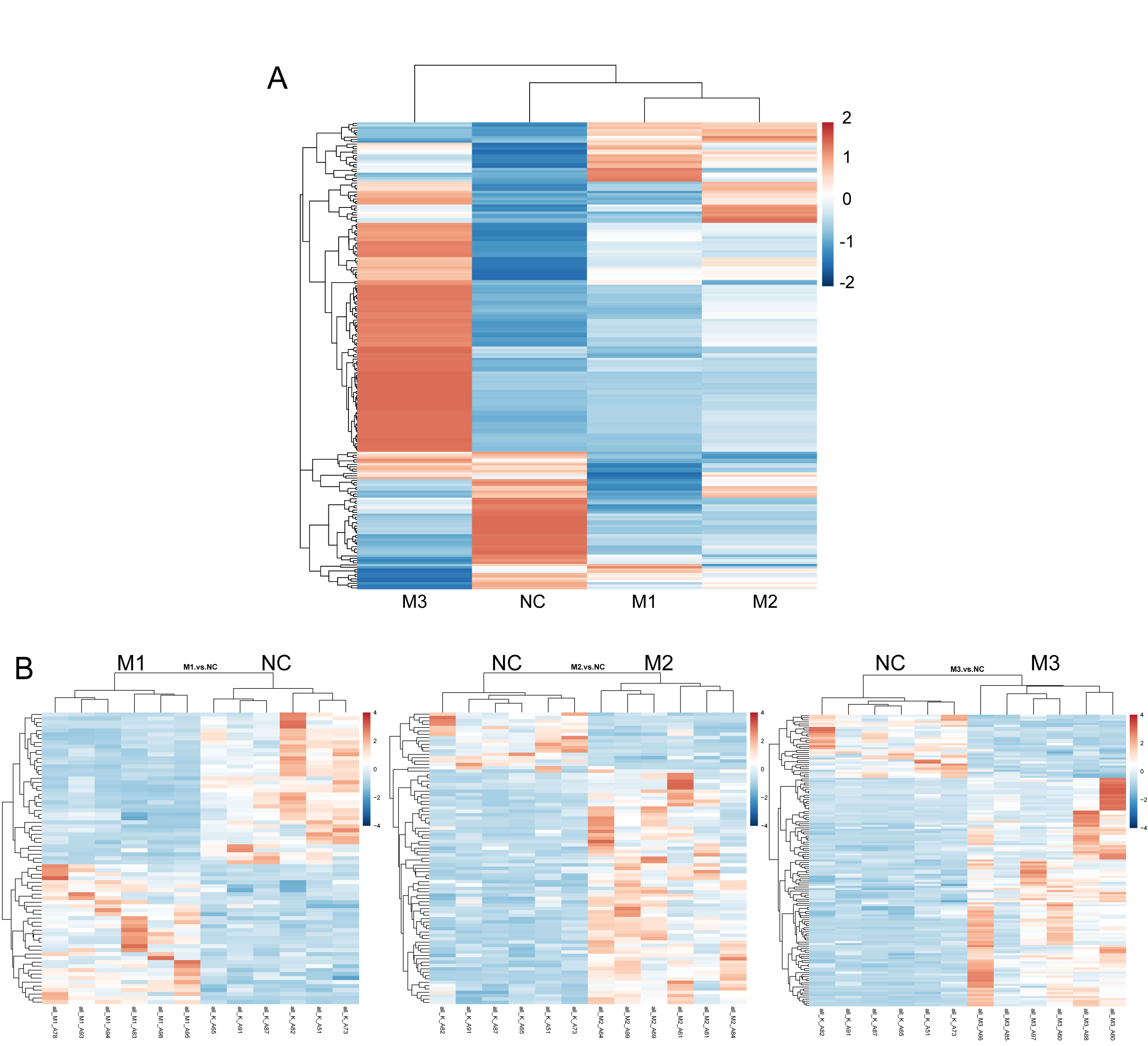

Supplement: Supplementary file 2 [file Image3.TIF]

| 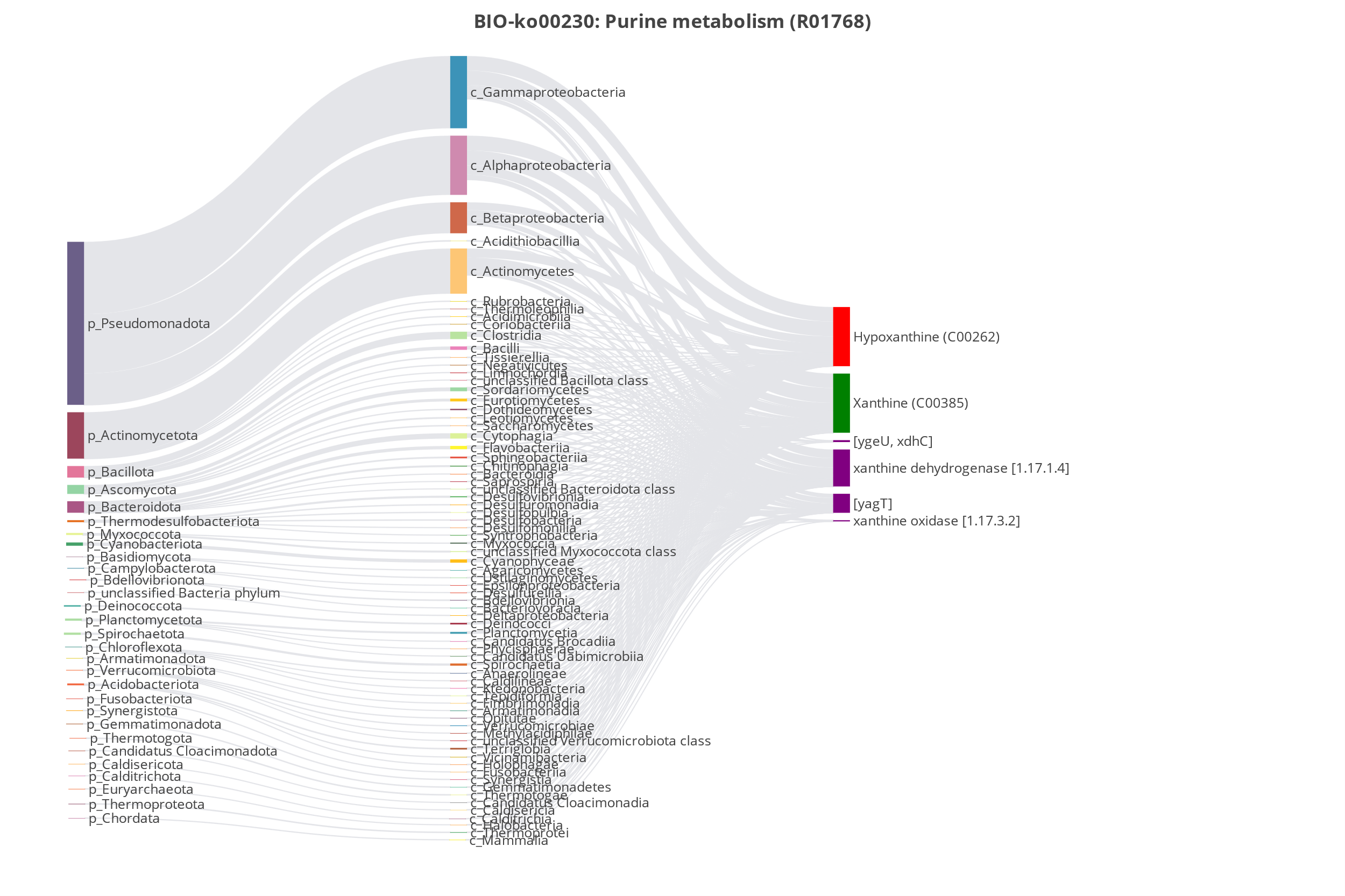 |
| --- |
| 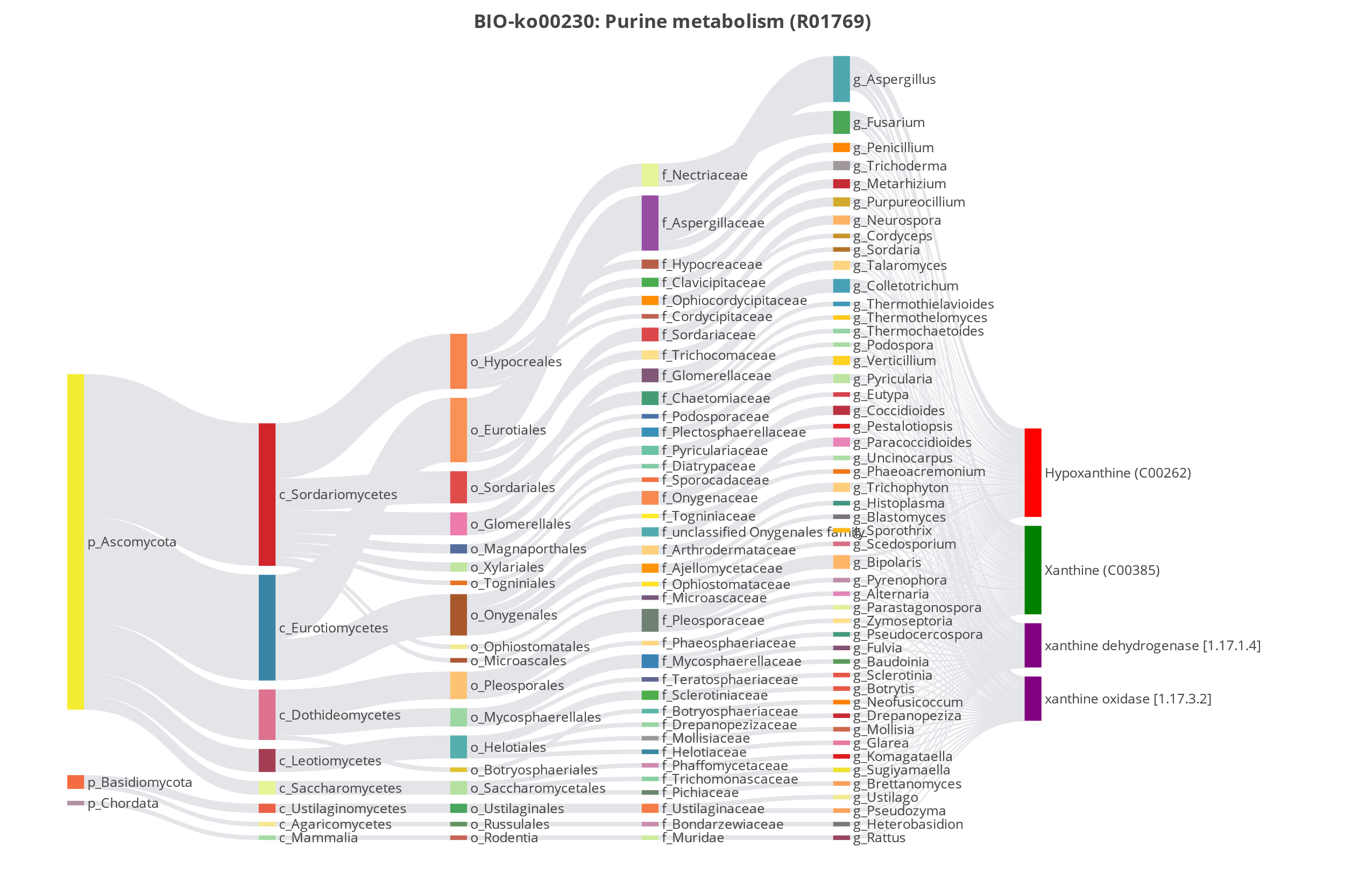 |
| 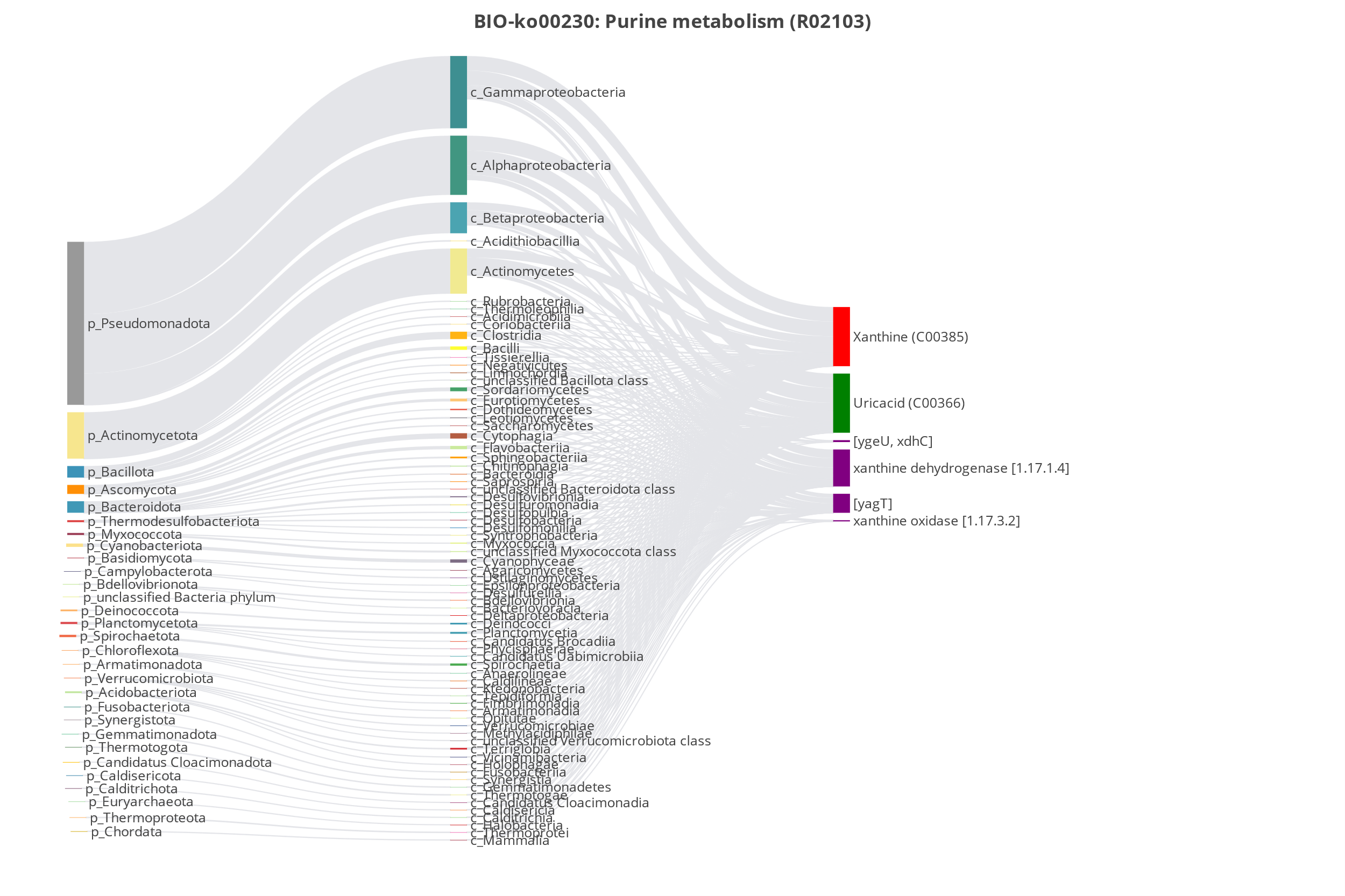 |
| 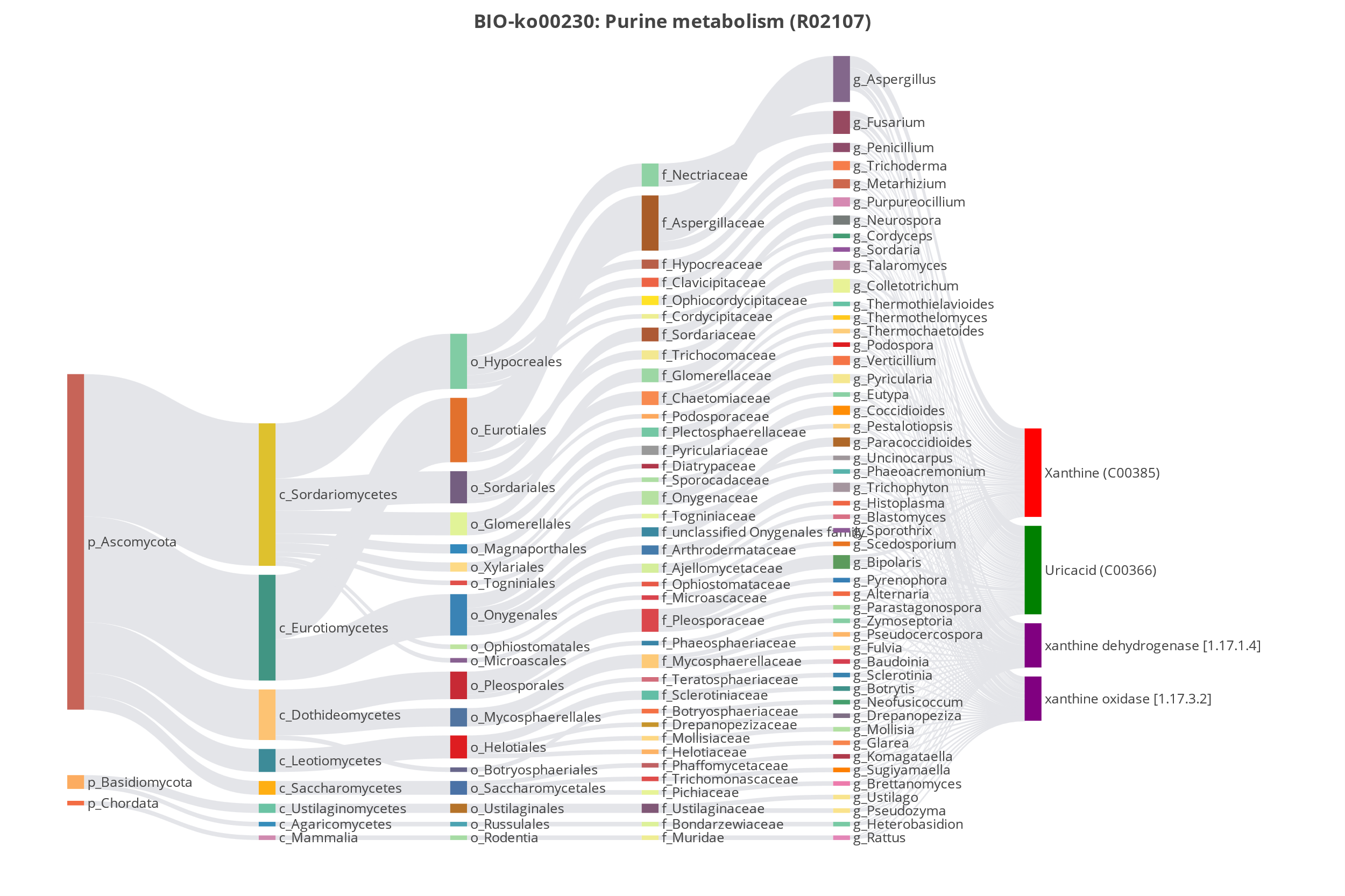 |

**Supplementary Fig. 4.** Sankey network of MetOrigin analysis.

Supplement: Supplementary file 3 [file DataSheet1.doc]

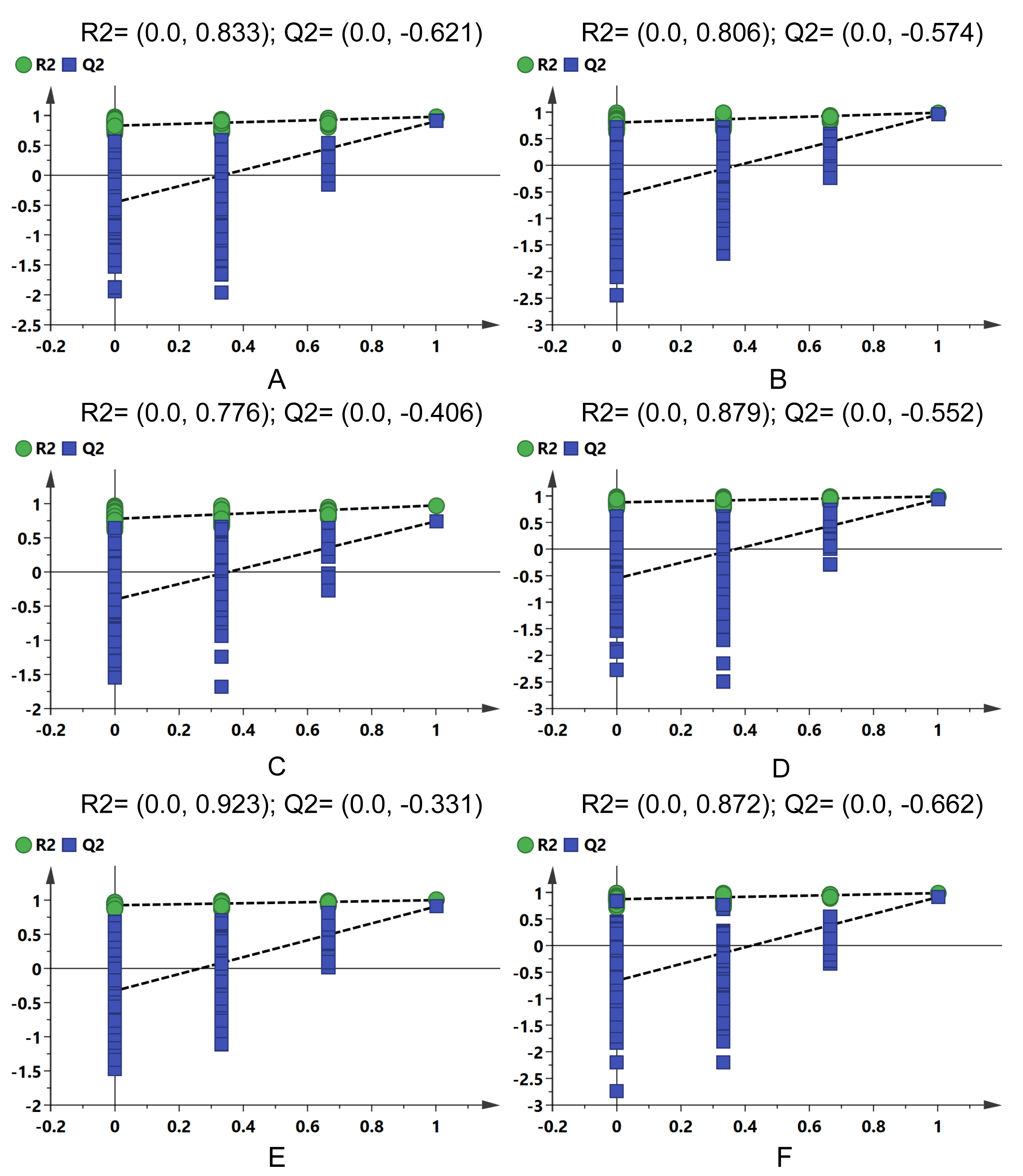

Supplement: Supplementary file 4 [file Image2.TIF]

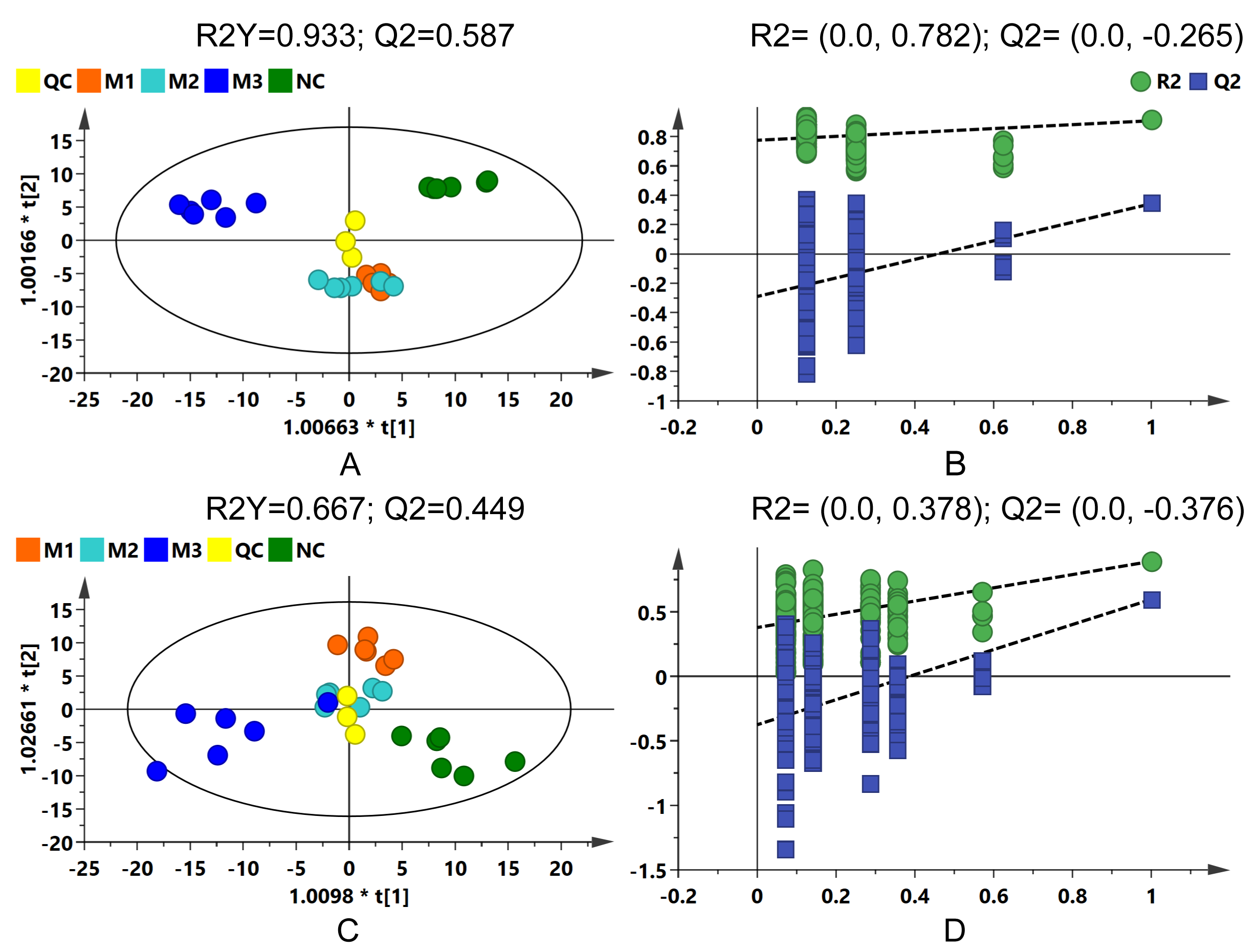

Supplement: Supplementary file 5 [file Image1.TIF]
